# Characterization of the interplay between DNA repair and CRISPR/Cas9-induced DNA lesions at an endogenous locus

**DOI:** 10.1038/ncomms13905

**Published:** 2017-01-09

**Authors:** Anne Bothmer, Tanushree Phadke, Luis A. Barrera, Carrie M Margulies, Christina S. Lee, Frank Buquicchio, Sean Moss, Hayat S. Abdulkerim, William Selleck, Hariharan Jayaram, Vic E. Myer, Cecilia Cotta-Ramusino

**Affiliations:** 1 Editas Medicine, 11 Hurley Street, Cambridge, Massachusetts 02141, USA

## Abstract

The CRISPR–Cas9 system provides a versatile toolkit for genome engineering that can introduce various DNA lesions at specific genomic locations. However, a better understanding of the nature of these lesions and the repair pathways engaged is critical to realizing the full potential of this technology. Here we characterize the different lesions arising from each Cas9 variant and the resulting repair pathway engagement. We demonstrate that the presence and polarity of the overhang structure is a critical determinant of double-strand break repair pathway choice. Similarly, single nicks deriving from different Cas9 variants differentially activate repair: D10A but not N863A-induced nicks are repaired by homologous recombination. Finally, we demonstrate that homologous recombination is required for repairing lesions using double-stranded, but not single-stranded DNA as a template. This detailed characterization of repair pathway choice in response to CRISPR–Cas9 enables a more deterministic approach for designing research and therapeutic genome engineering strategies.

## Abstract (web summary)

CRISPR-Cas9 has rapidly become a common molecular biology tool for modifying genomes and has been modified to generate single-strand nicks as well as double-strand breaks. Here the authors explore the DNA repair pathways activated by the different variants of Cas9.

The CRISPR–Cas9 system (clustered, regularly interspaced, short palindromic repeats/CRISPR-associated protein) has recently emerged as a method that enables robust and efficient genome engineering^1^,^2^. Initial *in vitro* experiments demonstrated that purified Cas9 protein of *Streptococcus thermophilus* and *Streptococcus pyogenes* could be used to drive the cleavage of DNA^3^,^4^, and that cleavage could be achieved using a chimeric single guide RNA (gRNA), only requiring the presence of a 5′-NGG protospacer adjacent motif (PAM)^4^. The Cas9/gRNA system was subsequently engineered to direct specific cleavage of target loci in eukaryotic cells, including human cells^5^,^6^. The cleavage activity of the Cas9 endonuclease is mediated through the coordinated activities of the HNH and RuvC catalytic domains^3^,^4^,^7^,^8^. While the HNH domain cleaves the gRNA complementary strand, cleavage of the non-complementary strand is mediated by the RuvC domain^4^. With both catalytic domains functional, Cas9 introduces a blunt double-strand DNA break (DSB), which is sensed by the cell's endogenous DNA repair system^1^.

Repair of DSBs is critical for the maintenance of genome stability. Organisms have evolved sophisticated DNA damage response pathways that sense the lesion and activate DNA repair to resolve the genomic insult^9^. The most active pathway in response to a DSB in mammalian cells is the canonical non-homologous end-joining (c-NHEJ) pathway, which repairs DSBs throughout the cell cycle resulting in either perfect repair or the formation of small insertions and deletions. However, a subset of DSBs will undergo endo- and exonucleolytic processing that exposes a 3′ overhang^10^, which is a suitable substrate for two possible mechanisms: alternative NHEJ (a-NHEJ), which is less dependent on sequence homologies^11^,^12^ and homology-directed repair (HDR), which relies on extensive homology^13^. Error-prone a-NHEJ is comprised of a variety of different and not fully characterized pathways that can lead to several outcomes, including deletions with or without microhomologies at the repair junctions, or insertions arising through annealing dependent pathways^12^,^14^. The HDR pathway comprises at least two sub-pathways: homologous recombination (HR) and single-strand annealing^15^. HR, a process that is highly regulated and typically error free, uses a sister chromatid, homologous chromosome or an ectopic site for repair in the S- and G2-phases of the cell cycle. Mechanistically, BRCA2 recruits the RAD51 recombinase to resected 3′-ends, which is required for homology search, strand invasion and ultimately the repair of the lesion^13^,^16^. Single-strand annealing, in contrast, mediates annealing between stretches of chromosome-internal homologies resulting in the loss of the intervening region, and is therefore considered an error-prone repair pathway^15^.

Point mutations that abrogate the catalytic activity of either Cas9 nuclease domain lead to the formation of Cas9 nickase mutants that introduce a single nick on the respective DNA strand^5^,^17^. Similar to DSBs, DNA nicks from exogenous sources or endogenous processes are sensed by high-fidelity single-strand break repair pathways^18^. However, if these nicks do not get repaired before S-phase, they can be transformed into DSBs by DNA replication. Cells then rely on DSB repair pathways, specifically scar-less repair through HR, for their resolution^19^,^20^,^21^. As a result, single nicks introduced by Cas9 variants only produce a low overall frequency of locus modification^17^,^22^,^23^,^24^. However, the use of Cas9 nickases with two gRNAs has been shown to increase the frequency of locus disruption similar to that of wild-type (WT) Cas9 levels, suggesting the generation of an intermediate structure more similar to a DSB^24^,^25^,^26^. Little is known, however, about the exact types of lesions paired nickases generate and which DNA repair processes are engaged as a result thereof.

In this study, we characterize the types of lesions generated by Cas9 variants and systematically examine the different DNA repair pathways engaged. We show that paired Cas9 nickases indeed generate DSBs with the predicted overhang lengths and polarities, resulting in the differential engagement of cell endogenous repair pathways, but only if the PAMs are facing outwards and not inwards. Furthermore, we provide evidence that the majority of the Cas9-induced single nicks at the target strand rely on RAD51 and BRCA2 for efficient and scar-less repair. Our study demonstrates that the CRISPR–Cas9 system serves as a tool to generate specific DNA structures at endogenous loci enabling the dissection of different repair responses. Our findings show that a detailed understanding of the repair processes engaged by the different Cas9-induced lesions is critical to successfully and efficiently achieve the desired genome-editing outcome.

## Results

### Cas9-induced lesions result in different repair outcomes

**Figure 1:**
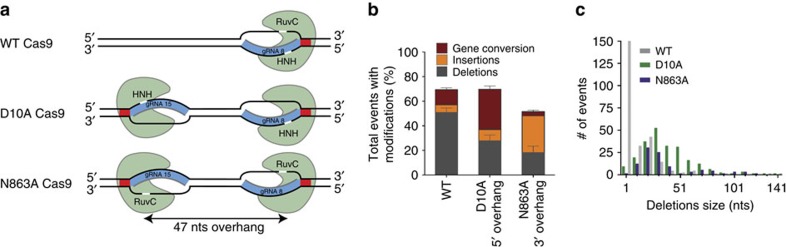
DNA lesions introduced by different Cas9 variants result in different repair outcomes. (**a**) Schematic of lesions introduced by either WT Cas9, D10A Cas9 or N863A Cas9 with gRNAs 8 and 15 (blue). The active nuclease domains are indicated for the respective mutants. PAMs are shown in red. The cut site is in white. (**b**) Overall modification frequency at the *HBB* locus separated into deletions, insertions and gene conversion events. The different repair outcomes after WT, D10A or N863A Cas9 activity in U2OS cells were measured by PCR amplification of the *HBB* locus, followed by Sanger sequencing of individual amplification products. Error bars were derived from five independent experiments. Data are represented as mean±s.e.m. Total number of sequences analysed was 569, 718 and 556 for WT Cas9, D10A Cas9 and N863A Cas9, respectively. (**c**) Plot of deletion size for WT (grey), D10A (green) and N863A (purple) Cas9 variants with gRNA 8 and 15 or only gRNA 8 in case of the WT Cas9. Individual deletion size data from each Cas9 variant were scored from Sanger sequencing and the total number of sequences analysed was 258, 241 and 103 for WT Cas9, D10A Cas9 and N863A Cas9, respectively.

Given its relevance as an important therapeutic target for genome editing, we studied the endogenous haemoglobin beta (*HBB*) locus. We identified a pair of gRNAs (designated 8 and 15), yielding similarly efficient locus disruption after WT *S. pyogenes* Cas9 DNA cleavage in U2OS cells. Using either gRNA 8 or gRNA 15 with Cas9 is predicted to result in the formation of a blunt DSB (Fig. 1a; Supplementary Fig. 1a). In addition, gRNAs 8 and 15 target opposite DNA strands at a distance of 47 nts with PAMs facing outwards with respect to each other (PAM-out) and are thought to result in DSBs with different polarities depending on the Cas9 variant used: the RuvC-mutant Cas9 (D10A) is predicted to yield a 5′ overhang (Fig. 1a), while Cas9 with an inactivated HNH domain (N863A) results in a 3′ overhang (Fig. 1a). These distinct structures are thought to have different propensities for engaging endogenous repair mechanisms: a 3′ overhang structure is a required HDR intermediate, but it is unclear whether a Cas9-induced 3′ overhang would indeed preferentially engage HDR. To address this question, we nucleofected U2OS cells with the respective Cas9 variants and gRNAs and examined the resulting genomic modifications with Sanger sequencing. DNA lesions generated by the three different Cas9 variants resulted in similar rates of overall modification at the *HBB* locus, suggesting a similar rate of DNA repair activity regardless of the Cas9-induced DNA lesion (Fig. 1b). No differences in overall expression levels were observed between the different Cas9 variants (Supplementary Fig. 1b). However, we observed striking differences in the types of repair outcomes (Fig. 1b) independent of the cell type (Supplementary Fig. 1c). To exclude the possible PCR bias of Sanger sequencing the *HBB* amplicon of a bulk population of cells, we isolated single-cell clones after treatment with different Cas9 variants and analysed their repair profile. We found that the repair profile distribution strongly resembles the distribution obtained with Sanger sequencing of a bulk population, and conclude that Sanger sequencing accurately represents repair outcomes and frequencies (Supplementary Fig. 1d).

**Figure 2:**
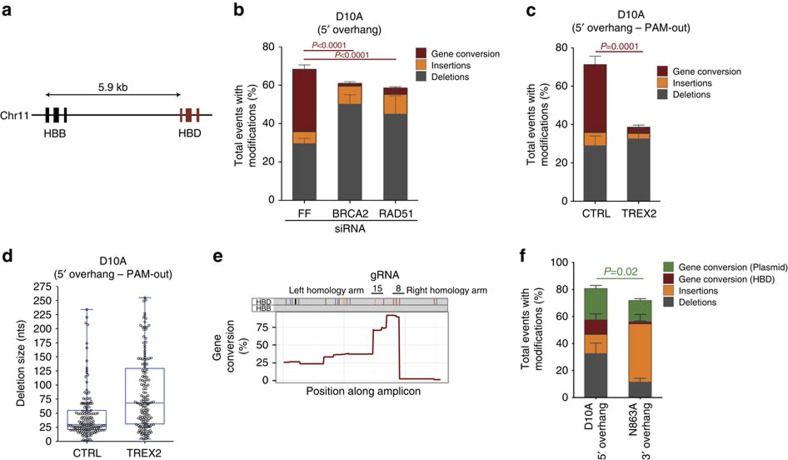
Characterization of repair outcomes of lesions introduced with the D10A Cas9 variant using gRNA pair 8/15. (**a**) Schematic illustration of the *HBB* and *HBD* loci on chromosome 11. (**b**) U2OS treated with an siRNA against firefly luciferase (control, labelled as FF), *BRCA2* or *RAD51* in addition to the D10A Cas9 and gRNAs 8/15. The overall modification frequency at the *HBB* locus resolved for deletions, insertions and gene conversion (GC) was determined by Sanger sequencing. *P* values (GC) were calculated using the two-tailed Student's *t*-test. Four independent experiments. Data are represented as mean±s.e.m. Total number of sequences analysed: 476, 407, 389 for siFF, siBRCA2 and siRAD51-treated cells. (**c**) The overall modification frequency resolved for deletions, insertions and GC scored by Sanger sequencing of the amplified *HBB* locus in U2OS cells expressing the D10A Cas9 and gRNAs 8/15 in the presence (TREX2) or absence (CTRL) of TREX2. Due to limiting amplicon size primer pairs underestimate deletion frequency. *P* value (GC) was calculated using the two-tailed Student's *t*-test. Five independent experiments. Total number of sequences: 578 for CTRL and 529 for TREX2. Data are represented as mean±s.e.m. (**d**) Scatter dot plot overlaid with a box and whisker plot representing the deletions size from Sanger sequences of U2SOS cells expressing D10A Cas9 with gRNAs 8/15 in the presence (TREX2) or absence (CTRL) of TREX2. Each individual dot represents one Sanger sequence with a deletion. Due to limiting amplicon size primer pairs underestimate deletion frequency. The line within the box depicts the median, with the box extending from the 25th to the 75th percentiles. The whiskers depict the minimum and maximum values of the data set. Five independent experiments, total number of sequences: 147 for CTRL and 149 for TREX2. (**e**) Graph showing positions of nucleotide mismatches (colours) and deletions (black) between the *HBD* and the *HBB* genes as coloured rods (top). Histogram of relative GC frequency plotted as a function of the position on the *HBB* locus (bottom). (**f**) Overall modification frequency at the *HBB* locus separated into deletions, insertions and GC from either the endogenous *HBD* gene or a plasmid source. The different repair outcomes after D10A or N863A Cas9 activity in U2OS cells followed by Sanger sequencing of individual *HBB* amplification products. Four independent experiments. Data are represented as mean±s.e.m. *P* value (Gene conversion (plasmid)) was calculated using the two-tailed Student's *t*-test. Total number of sequences analysed: 199 and 304 for D10A Cas9 and N863A Cas9.

These data suggest that different DNA ends generated indeed engage distinct DNA repair pathways. Specifically, WT Cas9-induced lesions were predominantly resolved as small deletions (median size=3 nts), while deletions observed when using D10A or N863A Cas9 dual nickases were larger (median size= 36 and 28 nts, respectively; Fig. 1c). Interestingly, 12.4% of total events after WT Cas9 editing harboured the footprint of the highly homologous delta globin (*HBD)* gene (defined as gene conversion (GC)). The *HBD* gene lies ∼5.9 kb downstream of *HBB* on chromosome 11 (Fig. 2a), and bears >90% sequence homology with respect to *HBB*. Due to the high degree of homology between these loci, we sequenced the *HBD* gene for off-target cleavage activity. While WT Cas9 with gRNA 15 yielded minor (<1%) off-target editing at the HBD locus, modifications <0.1% were detected for either WT Cas9 with gRNA 8 or dual nicking approaches (Supplementary Fig. 1e). GC from the endogenous *HBD* gene has previously been reported when *HBB* was targeted with gRNAs in human embryonic stem cells^27^. Surprisingly, given the presumed 5′ and 3′ respective overhangs, the rate of GC from *HBD* was significantly enhanced with D10A Cas9-induced lesions (32.8% in D10A versus 12.4% in WT; *P*=0.0001; two-tailed Student's *t*-test), while N863A Cas9-induced lesions showed a significant reduction in GC relative to WT Cas9 (3.5% in N863A versus 12.4% in WT; *P*=0.002; two-tailed Student's *t*-test; Fig. 1b). These results suggest that the presumed 5′ overhang DNA structure is particularly amenable to GC.

### GC proceeds through the HR pathway

GC is a highly precise mechanism that repairs DSBs during the S/G2 phases of the cell cycle through the HR pathway. To test whether D10A Cas9-induced GC proceeds through the HR pathway, we knocked down the core HR factors RAD51 and BRCA2 with short interfering RNAs (siRNAs), and found that both genes were required for efficient GC independent of the gRNA pair (Fig. 2b; Supplementary Fig. 2a). The finding that a 5′ overhang was more efficient in mediating GC than a 3′ overhang was surprising as an exposed single-stranded 3′ overhang is known to be a required HR intermediate. We hypothesized that the predicted 5′ overhang structure could be processed to yield a recombination favourable 3′ overhang. If this were the case, GC would be sensitive to an ectopically expressed 3′–5′ exonuclease such as TREX2 known to process 3′ overhangs^28^,^29^,^30^. Indeed, co-expression of TREX2 and D10A Cas9 resulted in a strong reduction in GC frequency, indicating that a 3′ overhang is part of the life cycle of a 5′ overhang undergoing GC independent of the gRNA pair (Fig. 2c; Supplementary Fig. 2b–d). In addition, we observed that on TREX2 expression the size of D10A Cas9-induced deletions significantly increased regardless of the guide pair used (Fig. 2d; Supplementary Fig 2e). We noticed that the overall modification frequency was reduced in TREX2-treated cells. As we observed the occurrence of long deletions in the presence of TREX2, we reasoned that our amplification strategy with primers separated by ∼600 nts on the *HBB* reference sequence could underestimate longer deletions. Increasing the amplicon size indeed captured even larger deletions (Supplementary Fig. 2f), suggesting that the apparent overall decrease in modification frequency observed on TREX2 expression could be due to assay limitations. In summary, these findings suggest that the 5′ overhang is processed into a 3′ overhang resulting in the exposure of a 3′ arm on the regions outside the gRNA 8 and 15 binding sites, which are then engaged in homology search. Interestingly, ∼ 30% of the GC events extend beyond the boundaries of the two nicking sites. The GC tracts are strongly asymmetric with the left homology arm preferentially converting to the *HBD* sequence (Fig. 2e), suggesting that the different arms differ in how they engage in homology search (Supplementary Fig. 2g). Moreover, the above difference suggests that mismatches in the homology arms might be a critical determinant of HR efficiency. The extent of mismatches differs between N863A and D10A Cas9-induced overhangs: the 3′ overhang of an N863A-induced DSB would harbour six mismatches with respect to *HBD,* while the D10A Cas9-induced 5′ overhang that is converted into a 3′ overhang would only contain a first mismatch after 37 or 47 nts depending on the arm (Supplementary Fig. 2g). To address whether this affects GC efficiency, we used a plasmid donor containing the endogenous *HBB* sequence with two point mutations as a surrogate GC substrate. This configuration would now disfavour homology search from the D10A Cas9-induced resected 3′-arm as there are two mismatches proximal to the 3′-end (positions 5 and 6), while no mismatches are present in the N863A Cas9-induced 3′ arm (Supplementary Fig. 2h). As shown in Fig. 2f, N863A-mediated GC from the plasmid using a 3′ protruding arm remains significantly less frequent than D10A Cas9-induced GC from a processed 5′ protruding arm. The difference, however, is less striking than for endogenous GC from the *HBD* locus, indicating that mismatches can result in rejection of homology arms.

### N863A Cas9-induced 3′ overhangs result in insertions

**Figure 3:**
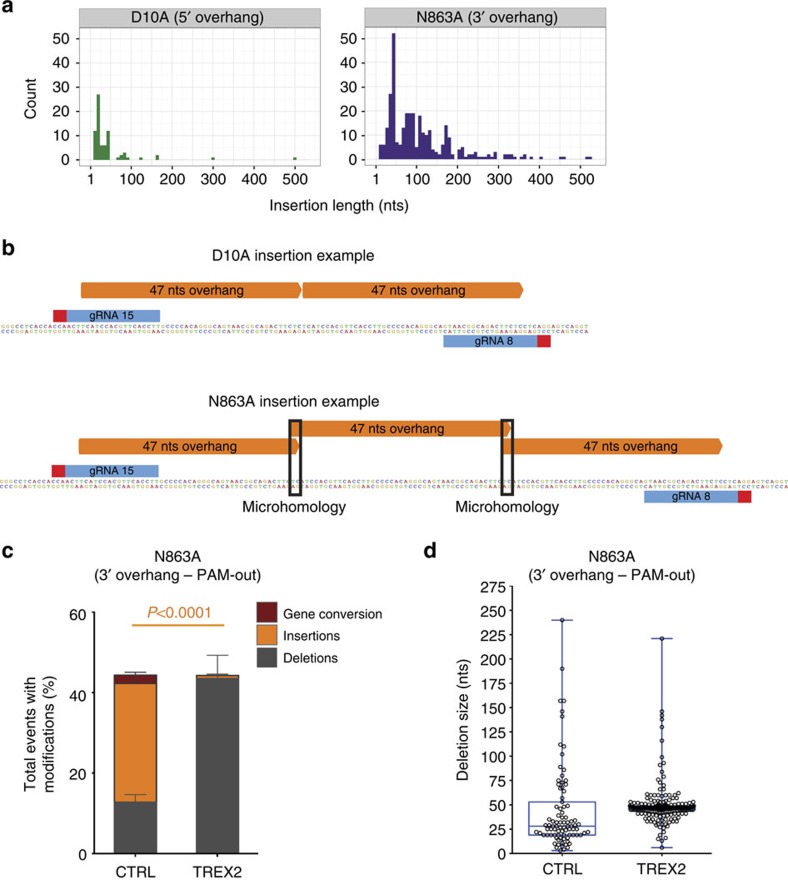
Characterization of insertions deriving from the D10A and N863A Cas9 variants using gRNA pair 8/15 in PAM-out orientation. (**a**) Histogram plot of insertion length for the D10A Cas9 (green) and N863A Cas9 (purple)-induced insertions. Individual insertion size data from each Cas9 variant was scored from Sanger sequencing data of U2OS cells treated with either D10A or N863A Cas9 and gRNA pair 8/15. Difference in length between D10A and N863A-induced insertions is significant: *P*=1.274 × 10^−12^ (permutation test). Number of insertions plotted is 75 for D10A and 325 for N863A. (**b**) Example of insertions of the full overhang resulting from N863A and D10A Cas9-induced lesions. Insertions (indicated as filled orange arrows) induced from D10A Cas9 do not show overlap while those from N863A Cas9 do indicating microhomology usage in the latter case (indicated in black box). Position of gRNA indicated in blue, with red box representing the PAM. (**c**) Overall modification frequency resolved for deletions, insertions and gene conversion scored by Sanger sequencing of the amplified *HBB* locus in U2OS cells expressing the N863A Cas9 variant and gRNA pair 8/15 in the presence (TREX2) or absence (CTRL) of TREX2. The *P* value for the difference in insertion frequency was calculated using the two-tailed Student's *t*-test. Data are represented as mean±s.e.m. The total number of sequences plotted from six independent experiments is 684 for CTRL and 672 for TREX2-expressing cells. (**d**) Scatter dot plot overlaid with a box and whisker plot representing the deletions size scored from Sanger sequencing data of U2SOS cells expressing the N863A Cas9 variant with gRNA pair 8/15 in the presence (TREX2) or absence (CTRL) of TREX2. Each individual dot represents one Sanger sequenced read harbouring a deletion. The total number of sequences plotted from six independent experiments is 87 for control and 264 for TREX2-expressing cells. The line within the box depicts the median, with the box extending from the 25th to the 75th percentiles. The whiskers depict the minimum and maximum values of the data set.

The predominant repair outcome of the predicted N863A-induced 3′ overhang structure was insertions (Fig. 1b: 29.9% for N863A compared with 9.0% for D10A). We noticed that almost all (98.5%) N863A Cas9-induced insertions were identical to all or part of the sequence found between the nicks, while only 71.5% of D10A Cas9-induced insertions could be mapped to the same sequence (Supplementary Fig. 3a). In addition, the N863A Cas9-induced insertions were significantly longer than in the D10A Cas9 condition (Fig. 3a), and we observed that N863A Cas9-induced insertions predominantly stem from the central part of the overhang. In contrast, D10A Cas9-induced insertions were mostly derived from the first 20 nts of the overhang structure (Supplementary Fig. 3b). Furthermore, we found that N863A Cas9-induced insertions more frequently contained several repetitions of the overhang structure, and in all but one case of full overhang repetition microhomology usage was evident (Fig. 3b; Supplementary Fig. 3a). In contrast, for the rare overhang repetition events observed in D10A Cas9-induced insertions no microhomology usage was evident (Fig. 3b; Supplementary Fig. 3a). Instead, we observed a complete duplication of the overhang suggesting that indeed a 5′ overhang structure is an initial outcome of a D10A-paired nick lesion. All of the above observations suggest a mechanistic difference between the N863A and D10A Cas9-induced insertions (see discussion).

To confirm the prediction that a 3′ overhang is generated and subsequently yields the observed insertions, we ectopically expressed TREX2 in the presence of N863A. We found that TREX2 expression almost completely abrogated the formation of insertions and instead observed a striking increase in deletion frequency (Fig. 3c). A percentage of 30.3 of TREX2-induced deletions were precise deletions of the overhang (47 nt), while no precise deletions were observed in control cells (Fig. 3d; Supplementary Fig. 3f). The increase in deletion frequency and the occurrence of precise deletions on TREX2 expression also held true for gRNA pairs with predicted overhang lengths of 37 or 61 nts (Supplementary Fig. 3c–f). These observations strongly suggest that the length and polarity of the substrate left by the D10A and N863A Cas9 is indeed as predicted: a 5′ and 3′ overhang, respectively, consisting of the nucleotides between the cleavage sites.

### PAM-in configuration strongly reduces strand separation

**Figure 4:**
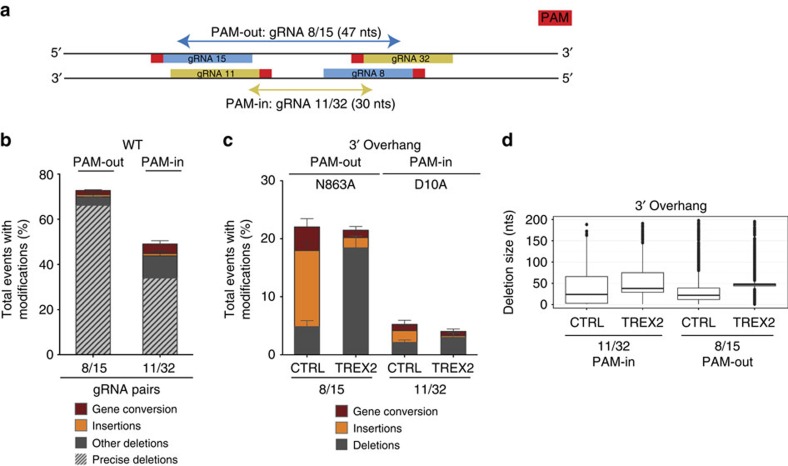
Paired nickase-induced lesions in the PAM-in orientation do not result in efficient strand separation and locus modification. (**a**) Schematic showing the position of gRNAs 8, 15, 11 and 32 on the HBB locus, alongside the predicted overhang length and PAM orientation (red). (**b**) Overall modification frequency resolved for deletions, insertions and gene conversion scored by Sanger sequencing of the amplified *HBB* locus in U2OS cells expressing the WT Cas9 variant and gRNA pairs 8/15 (PAM-out) or 11/32 (PAM-in). Data are represented as mean±s.e.m. Total number of sequences from four independent experiments is 324 for gRNA pair 8/15 and 280 for gRNA pair 11/32. (**c**) Overall modification frequency resolved for deletions, insertions and gene conversion scored by Sanger sequencing of the amplified *HBB* locus in U2OS cells expressing the N863A Cas9 variant and gRNA pair 8/15 (PAM-out) or the D10A Cas9 variant and gRNA pair 11/32 (PAM-in) in the presence (TREX2) or absence (CTRL) of the 3′–5′ exonuclease TREX2. gRNA expression was driven from PCR products containing the U6 promoter, gRNA sequence and TRACR (Methods). Data are represented as mean±s.e.m. The total number of sequences from three independent experiments is 203 for gRNA pair 8/15 CTRL, 572 for gRNA pair 11/32 CTRL, 224 for gRNA pair 8/15 TREX2 and 270 for gRNA pair 11/32 TREX2. (**d**) Box–whisker plot depicting the deletion size for either PAM-in gRNA pair 11/32 or PAM-out gRNA pair 8/15. Deletions were determined from sequences read by Illumina MiSeq. The line within the box depicts the median, with the box extending from the 25th to the 75th percentiles. The whiskers depict the minimum and maximum values within 1.5 inter-quartile range (IQR) of the median. Points with distance to median >1.5 IQR are shown individually.

All of the above experiments were performed with gRNAs in which the PAMs face outwards with respect to each other. Theoretically, 3′ and 5′ overhang structures could also be generated with inward facing PAMs (PAM-in). However, previous results^25^,^26^ indicated that only very limited gene editing could be observed with PAM-in gRNAs. We hypothesized that in a PAM-in orientation the two nicks might not result in strand separation and the formation of DSB-like intermediates. To address this question in our system we selected the gRNA pair 11/32 on the *HBB* locus with PAM-in configuration (Fig. 4a). We first confirmed that simultaneous cutting can occur with gRNA pair 11/32 by expressing both of these gRNAs with WT Cas9 in U2OS cells. Consistent with simultaneous occupancy and cleavage, we observed a high frequency of precise deletions between the predicted cleavage sites (Fig. 4b; Supplementary Fig. 4a). Using the PAM-in facing gRNA pair 11/32 with the D10A Cas9 variant would yield a 30 nt 3′ overhang. While the 3′ overhang generated with PAM-out gRNAs 8/15 in combination with Cas9 N863A resulted in the expected strand separation and apparent DSB formation, the PAM-in facing gRNA pair 11/32 expressed with D10A Cas9 did not result in a comparable degree of insertions or other locus disruption events (Fig. 4c). If a 3′ overhang was generated with the PAM-in gRNA pair 11/32, it would be susceptible to cleavage by the 3′–5′ exonuclease TREX2, resulting in precise deletions of the overhang sequence. Using Sanger sequence analysis, we detected no precise deletions for the PAM-in configuration. As the overall modification frequency was very low for PAM-in conditions, we subsequently used deep sequencing of repair events to analyse a larger number of deletions. We show that while precise deletions increased 83-fold in the presence of TREX2 for PAM-out gRNA 8/15 deletions, we only observed a fourfold increase for PAM-in gRNA 11/32 deletions (Fig. 4d). In addition, the gRNA pair 11/32 did not lead to efficient locus disruption rates when used with N863A Cas9, which would be expected to yield a 5′ overhang structure in a PAM-in configuration (Supplementary Fig. 4b). In summary, these data suggest that overhangs are formed infrequently with gRNAs in the PAM-in orientation.

### D10A Cas9-induced 5′ overhangs yield highest HDR rates

**Figure 5:**
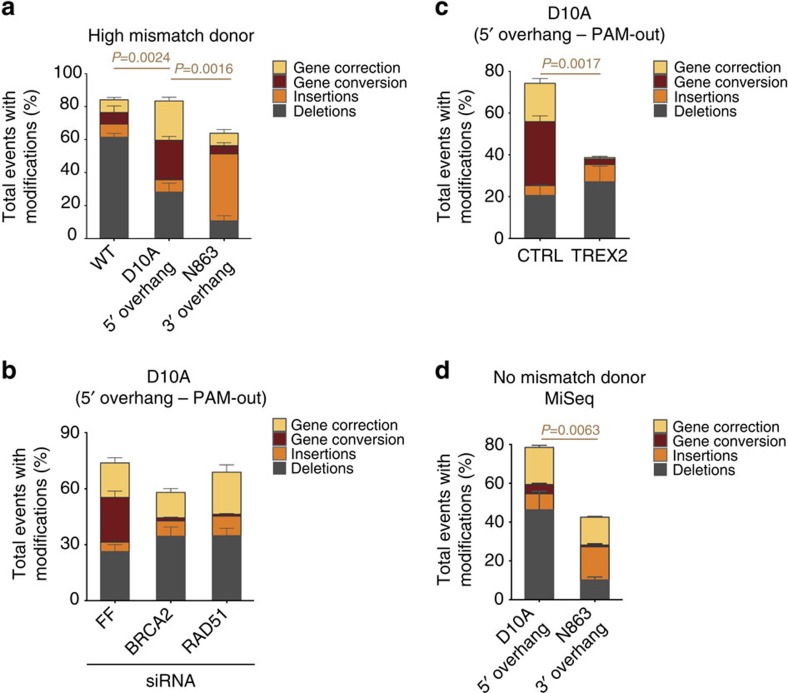
Characterization of gene correction using an ssODN. (**a**) Overall modification frequency at the *HBB* locus resolved for deletions, insertions, gene conversion (GC), and gene correction. The different repair outcomes after WT, D10A or N863A Cas9 activity in the presence of the ‘high mismatch donor' in U2OS cells were measured by Sanger sequencing of individual *HBB* amplification products. Data are represented as mean±s.e.m. The *P* values (gene correction) were calculated using the two-tailed Student's *t*-test. Total number of sequences plotted from three independent experiments: 200 for WT Cas9, 448 for D10A Cas9, and 422 for N863A Cas9. (**b**) Characterization of genetic requirements of gene correction. U2OS cells expressing an siRNA against control firefly luciferase (FF), BRCA2 or RAD51, in addition to D10A Cas9 and gRNAs 8/15 and the ‘high mismatch donor'. The overall modification frequency at the *HBB* locus resolved for deletions, insertions, GC and gene correction was determined by Sanger sequencing. Data are represented as mean±s.e.m. The total number of sequences from at least four independent experiments is 770 for FF, 652 for siBRCA2 and 657 for siRAD51-treated cells. (**c**) Overall modification frequency resolved for deletions, insertions, GC and gene correction scored by Sanger sequencing of the amplified *HBB* locus in U2OS cells expressing D10A Cas9, gRNAs 8/15 and the ‘high mismatch donor' donor in the presence (TREX2) or absence (CTRL) of TREX2. Data are represented as mean±s.e.m. The *P* value (gene correction) was calculated using the two-tailed Student's *t*-test. Total number of sequences from three independent experiments: 436 for CTRL and 338 for TREX2. (**d**) Overall modification frequency resolved for deletions, insertions, GC and gene correction using the ‘no mismatch donor' containing no mismatches for D10A or N863A Cas9 dual nick-induced, processed overhangs in U2OS cells. PCR amplification of the *HBB* locus was followed by MiSeq sequencing of individual amplification products and computational analysis to categorize repair events (see Methods). Data are represented as mean±s.e.m. The *P* value (gene correction) was calculated using the two-tailed Student's *t*-test.

To correct a specific genetic mutation, single-stranded oligodeoxynucleotides (ssODNs) harbouring the corrective sequence are frequently used (this type of repair is subsequently referred to as gene correction). We tested our different Cas9/gRNA variants for gene correction efficiencies using an ssODN that contains eight mismatches with respect to the reference sequence (‘high mismatch donor'). We found that the D10A Cas9-induced 5′ overhang yielded the highest gene correction rates (23.8% for D10A Cas9 compared with 7.7% for WT Cas9 (*P*=0.0024, two-tailed Student's *t*-test) and 7.5% for N863A Cas9 (*P*=0.0016, two-tailed Student's *t*-test); Fig. 5a).

To dissect the genetic requirements of the ssODN gene correction approach, we used siRNAs to knockdown the HR components BRCA2 and RAD51. Interestingly, we observed no change in correction frequency (despite elimination of GC) indicating that ssODN-mediated gene correction proceeds through a BRCA2/RAD51-independent pathway (Fig. 5b; Supplementary Fig. 5a). We asked whether similar to GC a 3′ intermediate is required for gene correction. When expressing TREX2 in cells undergoing gene correction with an ssODN we observed significantly reduced gene correction and GC demonstrating the requirement of a 3′ intermediate for both processes (Fig. 5c). While the frequency of gene correction was lower for WT and N863A Cas9-induced lesions than for D10A Cas9-induced lesions, we still observed a complete abrogation of gene correction in the presence of TREX2, indicating that a 3′ intermediate is also required for successful gene correction in these settings (Supplementary Fig. 5b). Since a processing step is required for the D10A-induced 5′ overhang to be converted into a 3′ overhang for gene correction to occur, the resulting protruding arms engaging in homology search have different degrees of mismatches when using the ‘high mismatch donor' ssODN (one mismatch on the 5′ arm that was converted to a 3′ arm versus five mismatches for the unprocessed N863A-induced 3′ overhang, Supplementary Fig. 5c). As this difference in mismatch could disfavour gene correction, we designed an ssODN with only two mismatches with respect to the reference *HBB* sequence, but with no mismatches with respect to the homology arms generated either by the N863A-induced 3′ overhang or the processed 5′ overhang induced by the D10A Cas9 (Supplementary Fig. 5c). Using this ‘no mismatch donor', we still observe that the 5′ overhang undergoes gene correction more frequently than the N863A-induced 3′ overhang independent of cell type (Fig. 5d). In this experiment, the apparent overall modification frequency for the N863A-induced nicks is lower than in previous examples (Fig. 1a; Fig. 5a). All previous experiments were performed using Sanger sequencing as a readout, while here the readout was high-throughput sequencing of the *HBB* amplicon with an Illumina MiSeq that underrepresents the insertion counts specifically for the N863A dual nicks. A detailed discussion of the difference between the sequencing methods and resulting consequences can be found in Supplementary Note 1; Supplementary Fig. 5d,e.

To ascertain that the differences in modification distribution observed are not locus dependent, we designed a paired nicking strategy using a gRNA pair with either the D10A or the N863A Cas9 variant targeting the *EMX1* locus. As for the *HBB* locus, a D10A-paired nicking approach resulting in a 5′ overhang yielded a higher frequency of gene correction from an ssODN than an N863A-paired nicking approach (25.5% for D10A and 9.3% for N863A; Supplementary Fig. 6a,b). Moreover, we noticed that similar to the *HBB* locus, the relative proportion of insertions is higher for N863A Cas9-induced 3′ overhangs. Again, similar to the *HBB* locus, the nature of the insertions in the N863A condition at the *EMX1* locus is different from the D10A Cas9-induced insertions: the N863A Cas9-induced insertions are significantly longer compared with the D10A-induced insertions (Supplementary Fig. 6c). They also display the characteristic usage of microhomology derived from the overhangs: 13% of long insertions (insertion length >35 nts) display signs of microhomology usage for N863A versus 2% of long D10A insertions (Supplementary Fig. 6c,d). In addition, similarly to the *HBB* locus, we observed that a D10A Cas9-induced 5′ overhang resulted in a complete duplication for 20% of long insertions, while this was only the case for 1% of N863A Cas9-induced insertions (Supplementary Fig. 6c,d).

In summary, we confirm that the different ends generated by the Cas9 variants engage different repair pathways at the *EMX1* locus similar to the *HBB* locus: D10A-induced 5′ overhangs result in higher levels of gene correction than N863A-induced overhangs, while the N863A-induced overhangs are predominantly resolved as insertions featuring microhomologies.

### D10A Cas9-induced single-nick repair is RAD51 dependent

**Figure 6:**
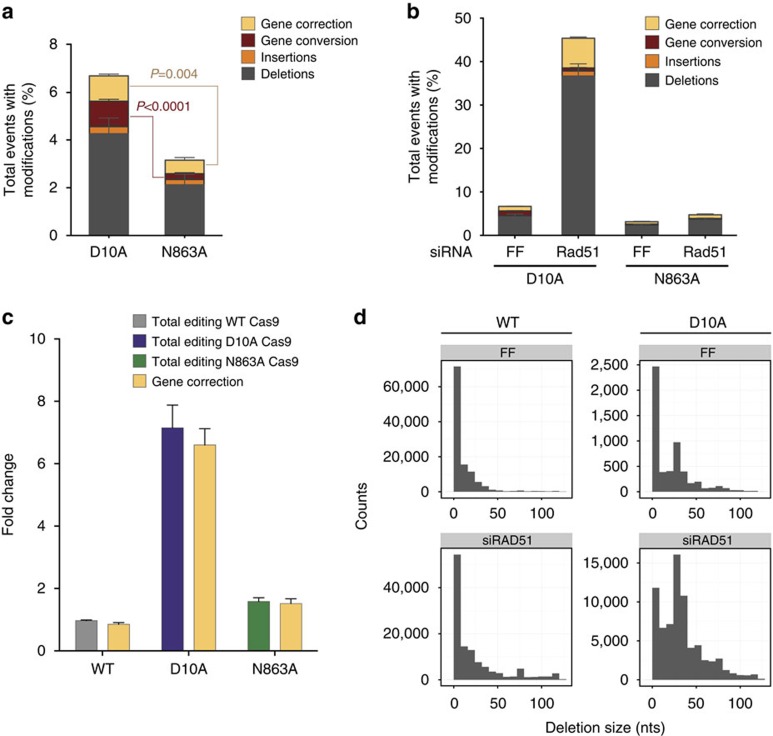
Characterization of repair pathway engagement of Cas9 variant-induced single nicks. (**a**) Frequency of deletions, insertions, gene conversion and gene correction (using the ‘high mismatch ssODN') from U2OS cells nucleofected with D10A Cas9 and N863A Cas9 and gRNA 8. Sequencing was performed using an Illumina MiSeq and modifications were quantified computationally (Methods). Three independent experiments. The *P* values for the difference in gene correction and gene conversion frequency was calculated using the two-tailed Student's *t*-test. Data are represented as mean±s.e.m. (**b**) Frequency of deletions, insertions, gene conversion and gene correction (using the ‘high mismatch ssODN') observed after U2OS cells were nucleofected with WT, D10A, or N863A Cas9 and gRNA 8, and an siRNA against RAD51 or firefly luciferase (FF) control. Sequencing was performed using an Illumina MiSeq and modifications were quantified computationally using (Methods). Three independent experiments. Data are represented as mean±s.e.m. (**c**) Bar graph of fold change in the rates of total editing events and gene correction (using the ‘high mismatch ssODN') for the D10A and N863A Cas9 mutants of cells treated with and siRNA against RAD51 relative to FF treated control. Three independent experiments. Data are represented as mean±s.e.m. (**d**) Representative histogram of the length of WT Cas9 and gRNA 8 or D10A Cas9 and gRNA 8-induced deletions in the presence or absence of RAD51.

As our results indicate that different DNA overhang structures produced by paired nickase strategies result in significantly different repair pathway engagement and repair outcomes, we sought to test whether similar effects could be observed with a single-nicking strategy. To this end we expressed gRNA 8 with either the D10A Cas9 nickase, which creates a single DNA nick in the target strand, or the N863A Cas9 nickase, which results in a DNA nick in the non-target strand, or a control WT Cas9 that yields a blunt DSB. The overall modification frequency compared with a WT DSB is strikingly reduced for both the D10A and N863A-generated single nicks in U2OS and K562 cells (81.3% for WT Cas9 compared with 6.7% for D10A Cas9 and 3.2% for N863A Cas9 for U2OS cells; Supplementary Fig. 7a,b), as previously reported^17^,^22^,^23^,^24^, suggesting the presence of an efficient single-nick repair mechanism. Moreover, when comparing the overall modification frequency of D10A and N863A Cas9-induced single-nick lesions, we observed that N863A Cas9-induced single nicks yielded approximately twofold fewer detectable repair events in U2OS with a less marked overall repair frequency difference in K562 cells (Fig. 6a; Supplementary Fig. 7b, respectively). Similar to the paired nicking approach, we observed that a D10A-induced single nick resulted in significantly higher ssODN-mediated gene correction and GC than a N863A-induced single nick (Fig. 6a; Supplementary Fig. 7b). One possible explanation could rest on differences in cutting efficiency between the D10A and the N863A Cas9 variants. We therefore performed *in vitro* DNA cleavage assays using different DNA substrates (Supplementary Fig. 7c). As previously shown for the equivalent nuclease mutants of *S. thermophilus* Cas9 (ref. 3), we did not detect significant differences in cutting efficiency between the D10A and N863A Cas9 variants using the same gRNA and target locus as in our experiments (Supplementary Fig. 7c,d). This suggests that either the repair of a N863A Cas9-induced single nick of the non-target strand proceeds more efficiently than the repair of the cleaved target DNA strand, or that the D10A-induced nick results in a more mutagenic intermediate (see Discussion).

Next, we tested whether GC and gene correction that result from single-nicking approaches proceed through similar pathways, as we described for the paired nickase approach: GC was sensitive to RAD51 knockdown while gene correction was not. Surprisingly, we observed a striking cell line independent sevenfold increase in the overall modification rate for D10A Cas9-induced nicks upon siRAD51 treatment (from 6.7% in FF control to 45.4% in siRAD51-treated U2OS cells, Fig. 6b,c and Supplementary Fig. 7e,f for K562 cells). This increase in overall modification was predominantly caused by a higher rate of deletions. In addition, we observed an approximately sixfold increase in gene correction on RAD51 knockdown for D10A Cas9-induced single nicks (from 1.1% in FF control to 6.9% in siRAD51-treated U2OS cells; Fig. 6b,c). Similar results were obtained with K562 cells (Supplementary Fig. 7e,f). While gene correction seems to have increased in absolute value in the RAD51 treated condition, there is no relative increase in gene correction efficiency taking the overall increase in modification frequency into account, suggesting that no selective increase for gene correction occurs in these conditions (Fig. 6c; Supplementary Fig. 7f). This differs from a previous report, in which only a selective increase in gene correction but not an increase in other modification events has been observed. This is likely due to differences in the assay methods: the use of reporter system with fluorescent read-out for only gene correction in contrast to our sequencing of all repair events^31^. In addition, on RAD51 knockdown, we observed that the deletion size was significantly increased for D10A Cas9-generated single nicks (Fig. 6d; *P*<2.2 × 10^−12^, permutation test). Interestingly, we were unable to observe a similar modification rate increase for the N863A Cas9-induced single nicks (Fig. 6b,c; Supplementary Fig. 7e,f) or the WT Cas9 control (Fig. 6c; Supplementary Fig. 7f). Lastly, to address whether our observations with respect to Cas9 single-nick processing are locus specific, we introduced a single nick using the D10A Cas9 variant at the *EMX1* locus. On siRNA-mediated knockdown of RAD51, we observed a striking increase in the overall modification rate mimicking the *HBB* results (Supplementary Fig. 7g). Altogether, the above observations suggest that D10A Cas9-induced nicks are mostly repaired by the HR machinery independent of the locus targeted. In the absence of functional HR these nicks are repaired by alternative, more error-prone pathways.

## Discussion

CRISPR–Cas9 is a highly versatile tool capable of generating a variety of DNA lesions that activate cell-intrinsic DNA repair mechanisms, which can be harnessed to drive gene disruption and gene correction approaches for genome engineering applications. In this study, we characterize the DNA lesions introduced by different Cas9 variants and elucidate the resulting cellular responses at the endogenous *HBB* locus.

**Figure 7:**
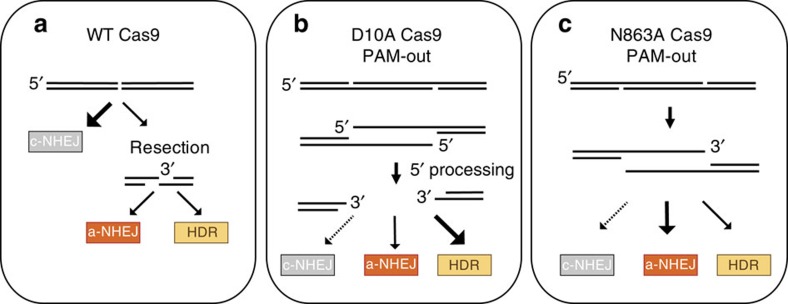
Models. (**a**) Model depicting the predominant engagement of c-NHEJ for the repair of WT Cas9 induced DSBs. (**b**) Model depicting the predominant engagement of HDR for the repair of D10A Cas9-induced DSBs with gRNAs in PAM-out configuration. (**c**) Model depicting the predominant engagement of a-NHEJ/SD-MMEJ and HDR for the repair of N863A Cas9-induced DSBs with gRNAs in PAM-out configuration.

Our findings indicate that the type of lesion generated is a critical determinant of repair pathway engagement and repair outcome. We confirmed that WT Cas9-induced DSBs are predominantly repaired through the c-NHEJ pathway (Fig. 7a), as previously reported^24^,^32^.

Using a paired nickase approach, we observe a shift in repair outcome distribution and a specific signature of repair outcomes for the different overhang structures. D10A-induced dual-nicking results in a 5′ overhang that more frequently engages HDR than N863A-induced 3′ overhangs (Fig. 7b). We provide evidence that GC, but not gene correction through an ssODN, is strictly dependent on the key HR components RAD51 and BRCA2. Repair using an ssODN has been recently referred to as single-strand template repair^33^, and our data show that single-strand template repair is a RAD51/BRCA2 independent pathway, in which the overhangs anneal to the ssODN homology regions to allow for repair (Supplementary Fig 8a). The finding that 5′ overhang structures yield higher levels of HDR than 3′ overhangs is counterintuitive, as 3′ overhangs are required for HR^15^. We show that the ectopic expression of TREX2 resulted in a marked reduction in HDR frequency, suggesting that exo- and/or endonucleolytic processing of the 5′ overhang structure occurs to generate an accessible 3′ end required for HDR (Supplementary Fig. 8b).

While 5′ overhangs also resulted in the formation of insertions, 3′ overhangs deriving from N863A Cas9 dual nicking led to insertions that were longer and originated almost exclusively from within the overhang sequence. Repetitions of overhang sequence were connected through junctional microhomologies, regardless of the locus targeted. Mechanistically, 3′ overhangs have been shown to engage translesion synthesis-dependent a-NHEJ pathways defined as synthesis-dependent microhomology-mediated end joining^34^,^35^ (Fig. 7c; Supplementary Fig. 8c). The observation that D10A-induced insertions are frequently derived from within the first 20 nts of the predicted overhang suggests a balance between 5′-end processing and 3′-dependent DNA ‘fill-in', likely utilizing the c-NHEJ pathway (Supplementary Fig. 8c). Moreover, the observation that a subset of insertions contain the full overhang suggests that a 5′ overhang structure of the predicted length is generated in the context of D10A-induced lesions (Supplementary Fig. 8c). Similarly, in the context of N863A-paired nickases the occurrence of perfect deletions of the overhang on TREX2 expression provides evidence that there is indeed the formation of a DSB with 3′ protruding arm that is equivalent to the distance between the nicks (Supplementary Fig. 8b).

In summary, we conclude that paired nicks with PAM-out orientation are indeed processed as DSBs, and that the different overhang polarities engage different repair pathways. The experiments supporting these conclusions were performed with paired nickases and gRNA pairs that orient the PAMs outwards. However, similar end structures can theoretically be achieved by selecting gRNA pairs for which the PAMs face inwards. In agreement with previous studies, we were unable to observe significant levels of locus modification using gRNA pairs with a PAM-in configuration^25^,^26^. We speculate that the inability to achieve significant locus modification is due to a topological barrier to strand separation (Supplementary Fig. 8d). The PAM directs Cas9 binding to the cleavage site, and while the gRNA invades the double helical DNA to form a gRNA–DNA hybrid with the target strand liberating the non-target strand, the PAM remains in a double-stranded configuration^17^. With two gRNAs in PAM-out configuration, the double-stranded PAM regions are external to the two gRNA–DNA hybrid structures, and the unwinding forces that act on the DNA are directed in opposite directions, which we speculate will favour strand separation. In a PAM-in configuration the gRNA-dependent unwinding occurs external to the two double-stranded PAMs, creating a double-stranded barrier to full strand separation (Supplementary Fig. 8d). This model predicts that cleavage events directed by paired nickases in the PAM-in configuration are sensed as two individual nicks.

Single DNA nicks are believed to be repaired by high-fidelity nick repair pathways. Here we provide a thorough characterization of how single nicks induced with the D10A or N863A Cas9 variant are repaired. N863A Cas9-induced single nicks showed fewer overall locus modification events than D10A Cas9-induced nicks, without an apparent difference in cleavage activity. Therefore, the finding that N863A single nicks lead to fewer modifications than D10A single nicks indicates a difference in repair pathway engagement that could be explained by enhanced accessibility of the non-target (N863A-nicked) strand^36^, which could lead to a more efficient repair of the N863A-induced nick (Supplementary Fig. 8e). A more mutagenic repair outcome for the D10A Cas9 nick could be due to the formation of the DNA–RNA hybrid between the gRNA and the target strand DNA (D10A-nicked), which could delay or complicate the repair of the nicked lesion on the target strand (Supplementary Fig. 8f). Additional evidence that repair differences between the nickase mutants exist comes from our RAD51/BRCA2 knockdown experiments, where we observe a striking increase in editing events only for D10A Cas9-induced single nicks, which are mostly mutagenic deletions. On the basis of these observations, we speculate that D10A Cas9-induced lesions might not be sensed until S-phase, where they are then converted into DSBs^19^,^20^,^21^. Mechanistically, this could be due to the inability of RAD51- and BRCA2-deficient cells to undergo replication fork remodelling to prevent replication fork run-off or undesirable processing at the nick, which has been described for more global type of damage^37^,^38^,^39^,^40^. Alternatively, the high degree of locus disruption could be due to the inability of RAD51/BRCA2-deficient cells to complete HR if run-off has occurred. While these hypotheses are not mutually exclusive, they would both result in increased conversion of DNA nicks to DSBs in S-phase where they would now be subject to the repair by alternative pathways (Supplementary Fig. 8f). Our observation of an increase in the size of the deletions in the absence of RAD51 could suggest the involvement of a-NHEJ pathways in the repair of these lesions in S-phase.

In conclusion, we have used the CRISPR–Cas9 system to introduce different DNA lesions at the endogenous *HBB* locus. We characterize the nature of the different CRISPR/Cas9-induced lesions and show that they engage a distinct set of repair pathways leading to differential DNA repair outcomes. In turn, these different Cas9-induced lesions allowed us to gain a better mechanistic understanding of repair pathway choice. This detailed characterization of Cas9-induced lesions at the endogenous *HBB* locus and their repair is an important step in unlocking the potential of genome engineering for human therapies.

## Methods

### gRNA selection and production

A list of gRNAs directing spCas9 were designed to target the human *HBB* locus requiring a NGG PAM. Potential gRNAs were aligned towards the human genome sequence to identify potential off-target sites with number of mismatches and indels. Variations in PAM sequence (NGG or NAG) were allowed for gRNAs with spacer lengths of 20 nucleotides. The target sequences are listed in Supplementary Table 2. Primers containing gRNA sequences were ordered using Integrated DNA Technologies. gRNAs 8, 15, 19 and 21 were generated by cloning annealed gRNA sequence oligos containing the target sequence into pMLM3636 (Addgene plasmid #43860), which contains a SpCas9-TRACR and a customizable U6-driven gRNA scaffold. The clones were maxi prepped and sequence confirmed by Sanger sequencing. gRNAs 8, 15, 11 and 32 for Fig. 4 and gRNAs 333, 334 and 335 for Supplementary Fig. 6 were generated by PCR and transfected as amplicons containing the U6 promoter, spacer sequence, and TRACR.

### Cell lines and cell culture

HEK293 (ATCC # CRL-1573), K562 (ATCC # CCL-243) and U2OS (ATCC #HTB-96) cells were maintained in Dulbecco's Modified Eagle Medium (Life Technologies) supplemented with 10% fetal bovine serum and 5% penicillin/streptomycin, and 2 mM Glutamax. Cells were kept at 37 °C in a 5% CO2 incubator. All the cell lines were routinely tested for mycoplasma contamination using a PCR-based approach.

### Nucleofection

Overall 250,000 cells were transfected using the Lonza 4D-Nucleofector (AAF-1002B 4D-Nucleofector Core unit, AAF-1002X 4D-Nucleofector X unit, SE Cell Line 4D-Nucleofector X Kit S V4XC-1032) with 200 ng of gRNA plasmid or a PCR product containing the U6 promoter/gRNA sequence/TRACR, and 750 ng of Cas9-variant plasmid (pJDS246-WT Cas9/pAF001-N863A Cas9/pJDS271-D10A Cas9 provided by K. Joung^41^), in the presence or absence of 50 pmol ssODN donor or 2 μg of plasmid DNA donor using the DN-100 program. For the ectopic expression of TREX2, 500 ng of plasmid AHB_26 (pcDNA3.1+TREX2-3XFLAG) was added to the Cas9/gRNA/ssODN plasmid mix before nucleofection. After nucleofection, the cells were allowed to incubate at room temperature for 10 min and then resuspended in complete Dulbecco's Modified Eagle Medium before transferring to six-well plates containing pre-warmed medium. The medium was changed 72 h after nucleofection and cells were collected 5 days post nucleofection. For sequences of ssODN or plasmid donors nucleofected see Supplementary Table 1.

### siRNA knockdowns

To knockdown BRCA2 and RAD51 the following siRNAs were used: FF-siRNA, Standard 0.015 μmol (CTM-127256), siGENOME Human BRCA2 (675) siRNA-SMARTpool, 5 nmol (M-003462-01-0005), siGENOME Human RAD51 (5888) siRNA-Individual, 5 nmol (D-003530-05-0005) and siGENOME Human RAD51 (5888) siRNA-Individual, 5 nmol (D-003530-07-0005). Specifically, immediately before nucleofection of 250K U2OS cells with Cas9 plasmids and gRNAs, 30 pmol of siBRCA2 or 15 pmol of both siRAD51 were added. Nucleofection was performed as described above. Samples were collected for gDNA extraction and further processing 5 days after nucleofections and knockdown efficiency was confirmed by western blot (Supplementary Methods).

### Genomic DNA extraction and PCR conditions

Genomic DNA was extracted from cells using the Agencourt DNAdvance genomic DNA isolation kit (Beckman Coulter Agencourt DNAdvance—Nucleic Acid Isolation from Mammalian Tissue #A48706) according to the manufacturer's directions. The CRISPR targeted genomic region of HBB was PCR amplified for subsequent Sanger sequencing using primers F1_HBB and R_HBB. Amplification was performed in a 50 μl reaction volume, consisting of 10 μl of 5 × Phusion HF buffer, 0.5 μM forward primer, 0.5 μM reverse primer, 200 μM dNTP, 1.5 μl DMSO, 0.5 μl of Phusion polymerase and 100 ng of gDNA template. PCR conditions were as follows: 30 s at 98 °C for initial denaturation, followed by 30 cycles of 10 s at 98 °C for denaturation, 15 s at 64 °C for annealing, 30 s at 72 °C for extension and 5 min at 72 °C for the final extension. For Supplementary Fig. 2f, we used primers HBB_primer_1_3F and HBB_primer_1_3R to amplify a longer region of the *HBB* locus for subsequent Sanger sequencing. The conditions were as above with 62 °C for annealing. To amplify the *HBD* locus to assay for off-target activity primers HBD_primer_F and HBD_primer_R were used with 64 °C for annealing. For primer sequences used see Supplementary Table 3.

### PCR purification

The PCR product was purified using (1.8 × ) Agencourt AMPure XP beads (Beckman Coulter Agencourt AMPure XP-PCR Purification #A63882) as per the manufacturer's protocol.

### Topo cloning and sequencing

The amplified locus fragments were then cloned into pCR4-TOPO vectors using the ZeroBlunt TOPO Cloning Kit (Life Technologies Zero Blunt TOPO PCR Cloning Kit for Sequencing with One Shot TOP10 Chemically Competent *E. coli* #K287540) and transformed in One Shot Top10 chemically competent *E. coli* cells. Cells were plated on Carbenicillin LB agar plates and incubated overnight at 37 °C. Plasmid DNA from 96 colonies per sample was sequenced by Macrogen Corp. and Genewiz, Inc. using an M13 forward or reverse primer.

### Illumina next-generation sequencing

Two rounds of PCR were performed on gDNA. To amplify the HBB locus HBB_Miseq_1F and HBB_Miseq_1R primers were used (Supplementary Table 3) and to amplify the EMX1 locus AHB_MiSeq_340 and AHB_MiSeq_341 primers were used. See Supplementary Table 3 for primer sequences.

Amplification was performed in a 50 μl reaction volume, consisting of 10 μl of 5 × HercII buffer, 0.5 μM forward primer, 0.5 μM reverse primer, 50 μM dNTP, 50 mM MgCl_2_, 0.5 μl of HercII polymerase and 250 ng of gDNA template. The thermal cycle was programmed for 2 min at 95 °C for initial denaturation, followed by 30 cycles of 20 s at 95 °C for denaturation, 20 s at 60 °C for annealing for HBB and 69 °C for EMX1, 20 s at 72 °C for extension and 3 min at 72 °C for the final extension.

Another round of PCR was performed to incorporate the P7 and P5 Illumina adapters and a unique 8-mer barcode sequence in a 50 μl reaction volume, consisting of 10 μl of 5 × HercII buffer, 0.5 μM forward primer, 0.5 μM reverse primer, 50 μM dNTP, 0.5 μl of HercII polymerase and 3 μl of round 1 PCR product. The thermal cycle was same as round 1 PCR with 25 cycles of denaturing, annealing and extension (see Supplementary Dataset 1 for primers).

The final PCR product was cleaned via AMPure (1.8 × ), eluted in 40 μl of H_2_O and size verified by electrophoresis on the Qiagen QIAxcel Advanced System. PCR product was then quantified using Picogreen, normalized into one library pool and sequenced on the Illumina Miseq according to the manufacturer's protocols.

### Quantification of editing events in NGS data

After demultiplexing, forward and reverse paired-end reads were merged to generate a single consensus sequence per read pair using PEAR v0.9.7 (ref. 42) with default settings. The resulting merged reads were aligned to the *HBB* amplicon sequence using needle, the Needleman–Wunsch algorithm implementation in the EMBOSS suite v6.6.0 (ref. 43). Insertions and deletions were identified by parsing the SAM files created by needle and parsing the CIGAR string^44^ for each read sequence. Reads with deletions that overlapped the ends of the amplicon that match the primer sequences were excluded from any further analysis (for example, to avoid having primer dimer confounded with reads from the amplicon).

To quantify GC and gene correction events in sequencing data, we first created pairwise alignments between the *HBB* amplicon sequence and the ssODN and homologous *HBD* sequences using needle. At each position in the alignment, we annotated whether single-nucleotide mismatches were present between the *HBB* sequence and either of the potential HDR templates. These positions in the *HBB* sequence can be therefore considered informative, as they allow discrimination between different types of HDR events. Reads from all samples were aligned to the *HBB* amplicon, as described above for the insertion and deletion analysis. At any position in which the DNA base reported in the read differed from the aligned base from the *HBB* sequence, the base was compared with the aligned sequences from *HBD* and the ssODN. Each read was then converted to a 3 × *L* binary matrix, where each column encodes whether the base in the read at a given position in the alignment matches the aligned sequences of *HBB, HBD* and/or the ssODN, where *L* is the number of bases in the read that were successfully aligned to *HBB* (‘M' status in the CIGAR string). The Viterbi algorithm was then used to identify segments of read sequences that matched the bases in the HDR templates instead of the sequence of the *HBB* locus. Intuitively, when two consecutive informative bases in the read were identified as matching either *HBD* or the ssODN sequence rather than the *HBB* sequence, the read was identified as having undergone GC or gene correction, respectively.

### Alignment of sanger sequencing reads for insertion analysis

To identify insertions in Sanger sequencing data, we used the Needleman–Wunsch algorithm implementation in the Biopython (pairwise2.globalms) package with the following parameters: base match score=2, base mismatch penalty=−1, gap open penalty=−50, gap extend penalty=−1. Insertion sequences were identified by taking the contiguous bases aligned to gap characters in the reference sequence.

### Overhang analysis

We identified the overhang sequence as located between the two predicted cleavage sites for each pair of gRNAs, assuming cleavage occurs three nucleotides 5′ of the start of the NGG PAM. For guides 8 and 15, the predicted overhang sequence is: TCATCCACGTTCACCTTGCCCCACAGGGCAGTAACGGCAGACTTCTC.

We classified insertion sequences according to whether they had no match to the overhang sequence, a partial match (at least one 9-mer in common) or a full match (complete overhang sequence contained within insertion sequence). We used Fisher's exact test to determine whether the proportion of sequences with partial or full overhang matches differed in a statistically significant manner, when using D10A or N863A nickases.

To identify segments of the overhang sequence that were overrepresented in insertions, we extracted all 4-mer sequences present in each insertion sequence and identified the positions where each 4-mer matched the overhang exactly. We then created histograms depicting the number of times that a particular 4-mer from the overhang was observed within insertion sequences. When a 4-mer was found multiple times within the overhang sequence, it was excluded from downstream analyses. We tested that the distribution of 4-mer positions was significantly shifted between D10A and N863A using a permutation test (permTS function from the ‘perm' R package with default settings).

### Data availability

The next-generation sequencing data can be accessed online at Sequence Read Archive under the BioProject accession code PRJNA353620. All other data generated during this study are available from the corresponding author on reasonable request.

## Additional information

**How to cite this article:** Bothmer, A. *et al*. Characterization of the interplay between DNA repair and CRISPR/Cas9-induced DNA lesions at an endogenous locus. *Nat. Commun.* **8,** 13905 doi: 10.1038/ncomms13905 (2017).

**Publisher's note:** Springer Nature remains neutral with regard to jurisdictional claims in published maps and institutional affiliations.

## Supplementary Material

Supplementary Information Supplementary Figures, Supplementary Tables, Supplementary Note, and Supplementary Methods.

Supplementary Data 1 This dataset lists the primers used for performing the second round of PCR for MiSeq library preparation.
