# Supplementary material for: Characterization of the interplay between DNA repair and CRISPR/Cas9-induced DNA lesions at an endogenous locus
Source: Nat Commun. 2017 Jan 9;8:13905. doi: 10.1038/ncomms13905 (PMC5227551; doi:10.1038/ncomms13905)
Supplement: Supplementary Information — Supplementary Figures, Supplementary Tables, Supplementary Note, and Supplementary Methods. [file ncomms13905-s1.pdf]

Supplementary Figure 1

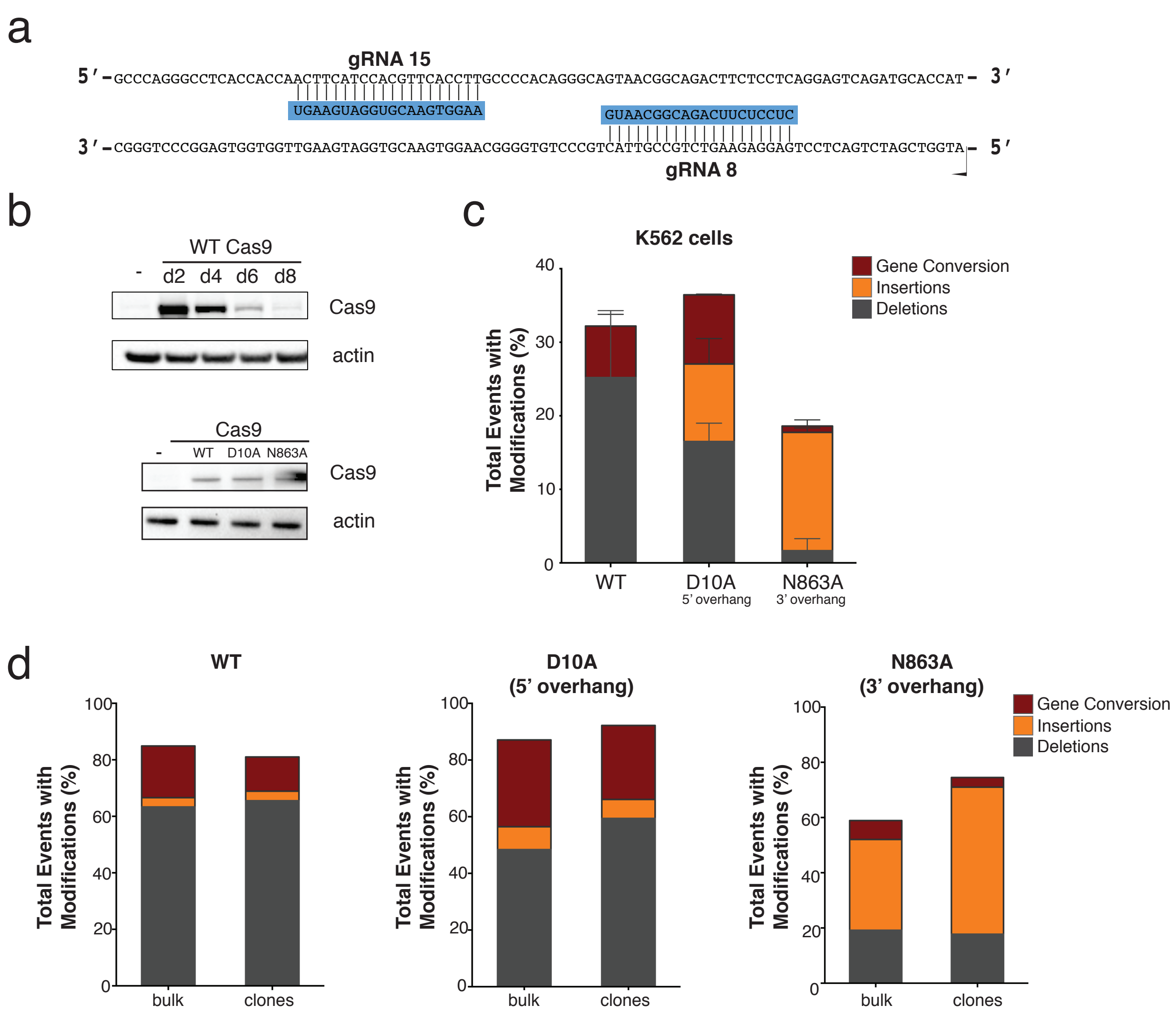

e

gRNA	Cas9	Modification HBD (%)
8	WT	0.04
15	WT	0.7
8+15	D10A	0.08
8+15	N863A	0.05

Supplementary Figure 1. Different DNA Lesions Introduced by Cas9 Variants Result in Different Repair Outcomes

a. Nucleotide sequence of the *HBB* locus and gRNAs 8 and 15 (blue). Arrow depicts the beginning of exon 1 of the *HBB* gene.

b. Western Blot for Cas9. Top: WT Cas9 expression time course over eight days. Bottom: Cas9 Western blot comparing expression level of the WT, D10A and N863A Cas9 variants on day 4.

c. Bar graphs showing the overall modification frequency separated into deletions, insertions, and gene conversion events for either WT Cas9, D10A Cas9, or N863A Cas9 nucleofected in K562 cells. Sanger sequencing. Data are represented as mean +/- SEM. Number of sequences analyzed was 91, 85 and 131 for WT Cas9, D10A Cas9, and N863A Cas9 respectively.

d. Bar graphs showing the overall modification frequency separated into deletions, insertions, and gene conversion events for either WT Cas9, D10A Cas9 or N863A Cas9 nucleofected bulk populations of cells (bulk) or individual single cell clones (clones). This experiment was performed because the PCR-based amplification step of a bulk population of cells with mixed repair events could in theory skew the repair profile. Sanger sequencing. Data are represented as mean. For WT Cas9 n=58 sequences were analyzed for bulk and n=92 clones were analyzed. For D10A Cas9 n=62 sequences were analyzed for bulk and n=81 clones were analyzed. For N863A Cas9 n=73 sequences were analyzed for bulk and n=96 clones were analyzed. The single cell clone analysis yielded a repair distribution very similar to the PCR analysis of the parental population of mixed cells (bulk).

e. Table showing the overall modification rate after either WT Cas9 or D10A and N863A dual nickases with gRNAs 8 and 15 at the off-target HBD locus determined by high throughput sequencing of HBD amplicons using Illumina MiSeq.

Supplementary Figure 2

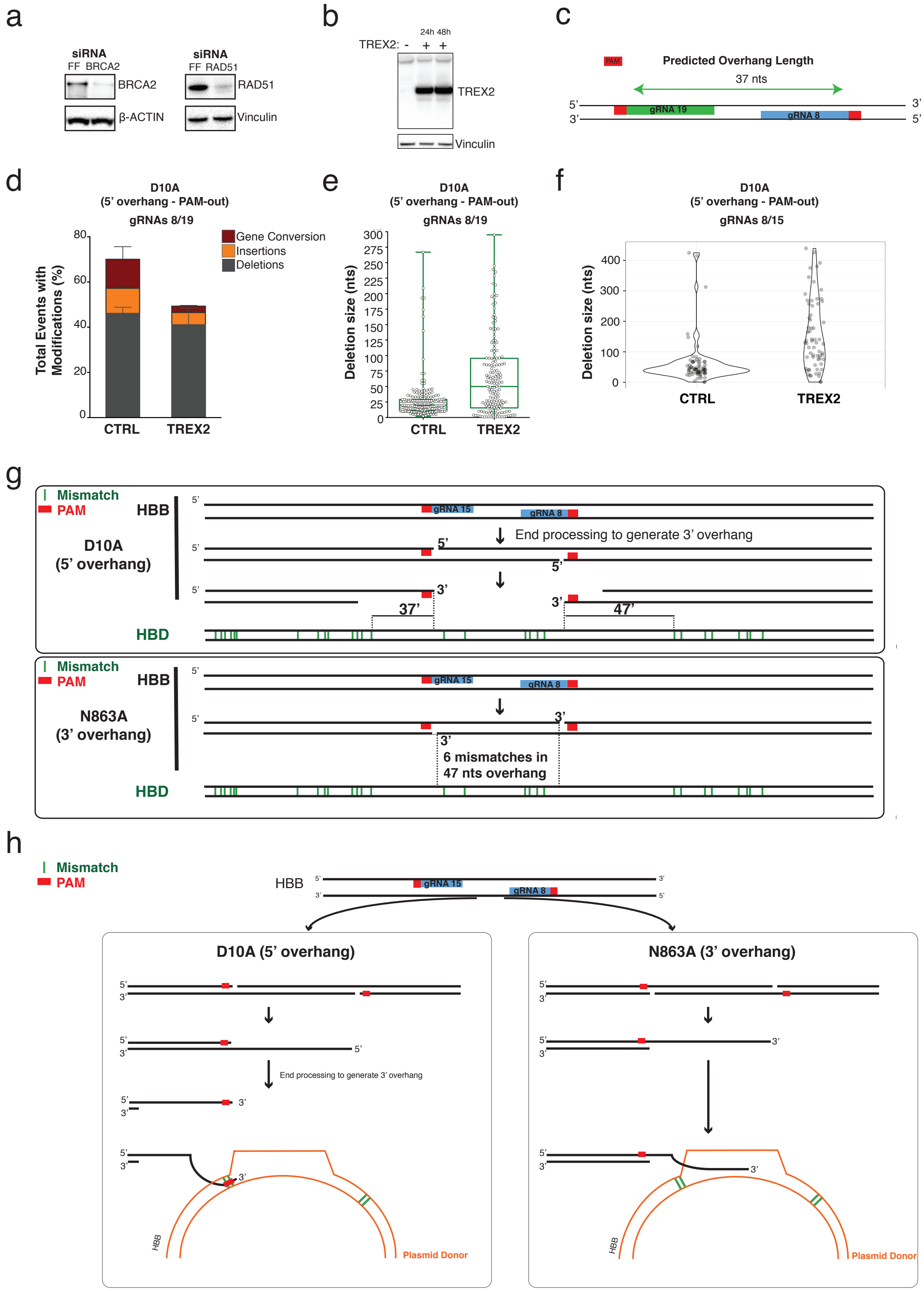

Supplementary Figure 2. Characterization of Repair Outcomes of Lesions Introduced with the D10A Cas9 Variant Using gRNA pair 8/19.

a. Western Blots controlling for knockdown efficiency after treatment of U2OS cells with siRNAs against firefly luciferase (FF), BRCA2, or RAD51. The loading control for BRCA2 knockdown is β–ACTIN, and the loading control for RAD51 knockdown is VINCULIN.
b. Western Blot controlling for expression of TREX2 in U2OS cells. TREX2 is expressed from pcDNA3.1 and assayed 24h and 48h after nucleofection as indicated. Loading control is VINCULIN.
c. Schematic showing the position of gRNAs 8 (blue) and 19 (green) on the *HBB* locus alongside the predicted overhang length. PAMs are shown in red.
d. Overall modification frequency resolved for deletions, insertions, and gene conversion events scored by Sanger sequencing of the amplified *HBB* locus in U2OS cells expressing the D10A Cas9 variant and gRNA pair 8/19 in the presence (TREX2) or absence (CTRL) of the 3'-5' exonuclease TREX2. Sanger sequencing. Primer pairs with an amplicon size that is underestimating the deletion frequency (due to inability to detect long deletions) were used due to technical limitations. Data are represented as mean +/- SEM. Numbers of sequences analyzed in three independent experiments is 422 for CTRL and 396 for TREX2.
e. Scatter dot plot overlaid with a box and whisker plot representing the deletion size scored from Sanger sequencing data of U2SOS cells expressing the D10A Cas9 variant with gRNA pair 8/19 in the presence (TREX2) or absence (CTRL) of TREX2. Each individual dot represents an Sanger sequenced read harboring a deletion. Primer pairs with an amplicon size that is underestimating the deletion frequency (due to inability to detect long deletions) were used due to technical limitations. The total number of sequences plotted from three independent experiments is 198 for CTRL and 161 for TREX2 expressing cells.
f. Scatter dot plot overlaid with a violin plot representing the deletion sizes scored from Sanger sequencing data of larger amplicons (see Methods for primer details) of U2SOS cells expressing the D10A Cas9 variant with gRNA pairs 8/15 in the presence (TREX2) or absence (CTRL) of TREX2. Each individual dot represents one Sanger sequenced read

harboring a deletion. A total of 85 deletions are plotted for CTRL, and 67 deletions are plotted for the +TREX2 condition.

g. Scheme displaying the mismatches with respect to the HBD locus of Cas9 generated overhangs. Top: D10A-generated 5' overhang is processed into a 3' overhang. Bottom: N863A-generated 3' overhang.

h. Schematic of the plasmid DNA donors that bear homology to the HBB locus. PAMs in the plasmid are mutated to prevent cleavage of the plasmid. The different degrees of locus processing after either D10A (left) or N863A (right) dual nicking is schematically depicted, along with the resulting homology arm undergoing homology search using the plasmid donor. While there are no mismatches in the N863A-induced 3' overhang with respect to the plasmid, the homology arm as a result of D10A induced cleavage contains two mismatches at the tip of the arm.

Supplementary Figure 3

a

	D10A	N863A	p-value (D10A v. N863A)
# Insertions deriving from overhang (% of all)	75 (71.5%)	325 (98.5%)	1.022×10^{-15}
# Insertions with overhang duplication (% of all)	6 (5.7%)	1 (0.3%)	0.001
# Insertions with microhomology usage (% of all)	0 (0%)	66 (20.0%)	<0.0001
# Insertions analyzed	105	330	

p value: Fisher's exact test

b

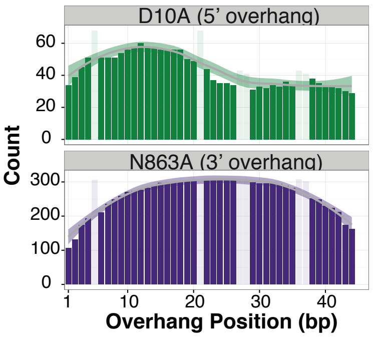

c

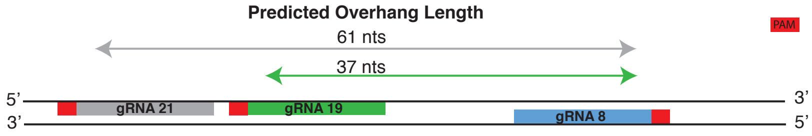

d

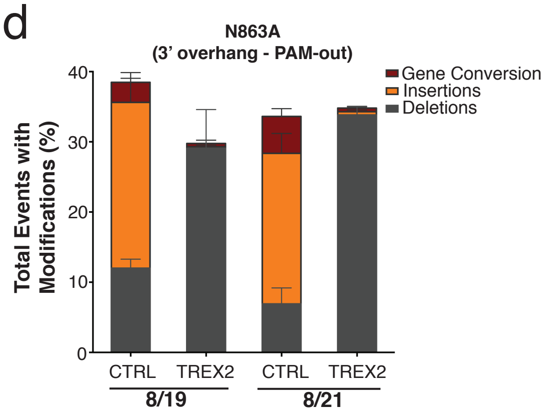

e

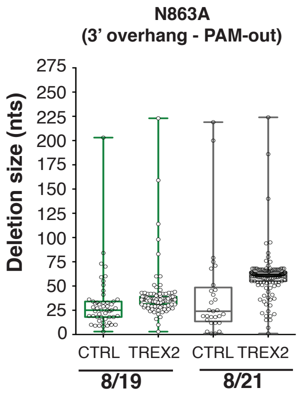

f

gRNA Pair	Predicted Overhang (nts)	Seqs with precise overhang deletions (%)	Seqs with overhang deletions within 5 nts (%)	
8/15	47	0	4.6	CTRL
		30.3	59.1	TREX2
8/19	37	0	3.9	CTRL
		12.1	44.0	TREX2
8/21	61	0	0	CTRL
		23.2	43.5	TREX2

Supplementary Figure 3. Characterization of Insertions Deriving from the D10A and N863A Cas9 Variants Using gRNA pairs 8/15, 8/19, and 8/21.

a. Table indicating the number and percentage of insertions from N863A and D10A Cas9-induced lesions that are derived from the overhang and that contain the full overhang repetition. Sequences that had at least a consecutive stretch of 9 nts of the predicted overhang sequence were considered to be derived from the overhang. The p-value was calculated using Fisher's exact test.
b. Histogram plot showing how frequently 4-mers found within insertion sequences aligned to different positions along the overhang sequence for guides 8 and 15 directed D10A Cas9 (green) and N863A Cas9 (purple) variants. Light purple or light green indicate positions in the overhang with repeated 4-mers. Parts of insertions sequences containing these repeated 4-mers cannot be uniquely mapped to a single position within the overhang.
c. Schematic showing the position of gRNAs 8 (blue), 19 (green), and 21 (grey) on the *HBB* locus, alongside the predicted overhang length. PAM orientation in red.
d. Overall modification frequency resolved for deletions, insertions, and gene conversion scored by Sanger sequencing of the amplified *HBB* locus in U2OS cells expressing the N863A Cas9 variant and gRNAs 8/19 or 8/21 in the presence (TREX2) or absence (CTRL) of TREX2. Data are represented as mean +/- SEM. The number of sequences analyzed in three independent experiments is 427 for gRNA pair 8/19 CTRL, 376 for gRNA pair 8/19 TREX2, 445 for gRNA pair 8/21 CTRL, and 407 for gRNA pair 8/21 TREX2.
e. Scatter dot plot overlaid with a box and whisker plot representing the deletion sizes scored from Sanger sequencing data of U2SOS cells expressing the N863A Cas9 variant with gRNA pairs 8/19 or 8/21 in the presence (TREX2) or absence (CTRL) of TREX2. Each individual dot represents one Sanger sequenced read harboring a deletion. The number of deletions analyzed in three independent experiments is 51 for gRNA pair 8/19 CTRL, 116 for gRNA pair 8/19 TREX2, 29 for gRNA pair 8/21 CTRL, and 128 for gRNA pair 8/21 TREX2.

f. Table showing the percentage of deletions that have the precise predicted overhang deleted and the percentage of deletions that fall within a range of +/- 5 nts for N863A Cas9-induced lesions with gRNA pairs 8/15, 8/19, or 8/21 in the presence or absence of TREX2.

Supplementary Figure 4

a

gRNA Pair	Predicted Deletion size (nts)	Precise overhang deletions (% of deletions)
8/15	47	94.5
11/32	30	77.7

b

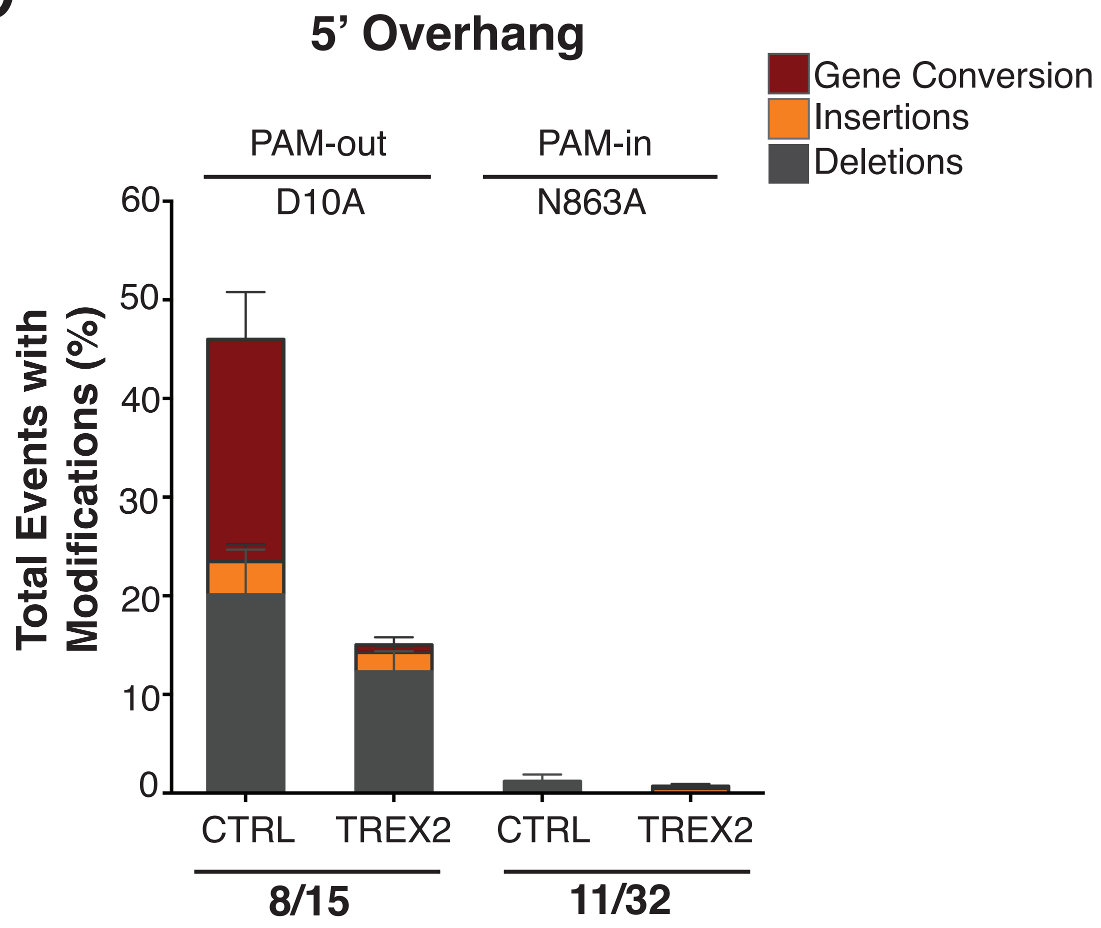

Supplementary Figure 4. Paired Nickase-Induced Lesions in the PAM-in Orientation do not Result in Efficient Strand Separation and Locus Modification.

a. Table showing the percentage of deletions that harbor a precise deletion between the two Cas9 WT cuts scored by Sanger sequencing of the amplified *HBB* locus in U2OS cells expressing the WT-Cas9 variant and gRNA pairs 8/15 (PAM-out) or 11/32 (PAM-in) from four independent experiments.

b. Overall modification frequency resolved for deletions, insertions, and gene conversion scored by Sanger sequencing of the amplified *HBB* locus in U2OS cells expressing the D10A Cas9 variant and gRNA pair 8/15 (PAM-out) or the N863A Cas9 variant and gRNA pair 11/32 (PAM-in) with (TREX2) or without (CTRL) the expression of 3'-5' exonuclease TREX2. Data are represented as mean +/- SEM. Total number of sequences from three independent experiments is 198 for gRNA pair 8/15 CTRL, 192 for gRNA pair 8/15 TREX2, 247 for gRNA pair 11/32 CTRL, and 199 for gRNA pair 11/32 TREX2.

Supplementary Figure 5

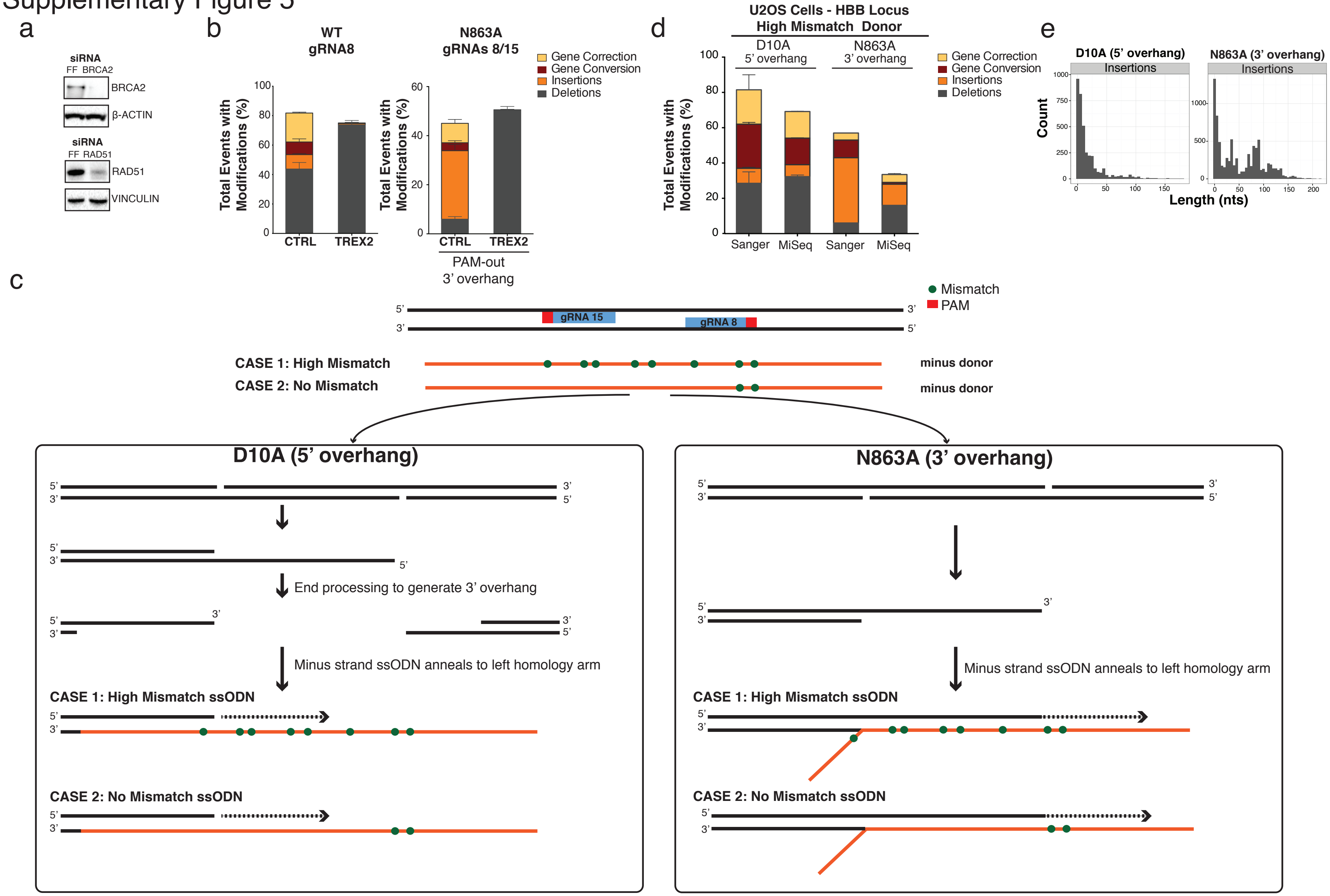

Supplementary Figure 5. Characterization of Gene Correction Using a Single-Stranded Deoxyoligonucleotide Donor.

a. Western Blots controlling for knockdown efficiency after treatment of U2OS cells with siRNAs against firefly luciferase (FF), BRCA2, or RAD51. The loading control for BRCA2 knockdown is β–ACTIN, and for RAD51 knockdown VINCULIN.

b. Overall modification frequency resolved for deletions, insertions, gene conversion, and gene correction scored by Sanger sequencing of the amplified *HBB* locus in U2OS cells expressing the WT Cas9 variant with gRNA 8, or the N863A Cas9 variant with gRNA pair 8/15 in the presence (TREX2) or absence (CTRL) of TREX2. Data are represented as mean +/- SEM. The total number of sequences from three independent experiments is 394 for WT gRNA8 CTRL, 434 for WT gRNA8 TREX2, 448 for N863A gRNAs 8/15 CTRL, and 341 for N863A gRNAs 8/15 TREX2.

c. Schematic showing the ssODN “high mismatch donor” donor containing eight mismatches with respect to the HBB locus (CASE 1) or the “No mismatch donor” (CASE 2) containing two mismatches with respect to the HBB locus. Differential processing of the D10A and N863A induced 5’ and 3’ arms lead to various mismatches in the homology arms with respect to the donor sequence for the “high mismatch donor”, but to no mismatches in either D10A or N863A-induced homology arms for the “no mismatch donor”.

d. Overall modification frequency resolved for deletions, insertions, gene conversion, and gene correction scored by either Sanger sequencing or Illumina MiSeq of the amplified *HBB* locus in U2OS cells expressing D10A or N863A Cas9 with gRNAs 8 and 15. Data are represented as mean +/- SEM. A total of 137 and 70 sequences were analyzed by Sanger sequencing for D10A and N863A. A total of four and two samples were subjected to MiSeq analysis for D10A and N863A, respectively.

e. Left: Representative histogram plot of the deletion size after D10A dual nicking with gRNAs8 and 15 determined by Illumina MiSeq analysis. Right: Representative histogram plot of the deletion size after N863A dual nicking with gRNAs8 and 15 determined by Illumina MiSeq analysis.

Supplementary Figure 6

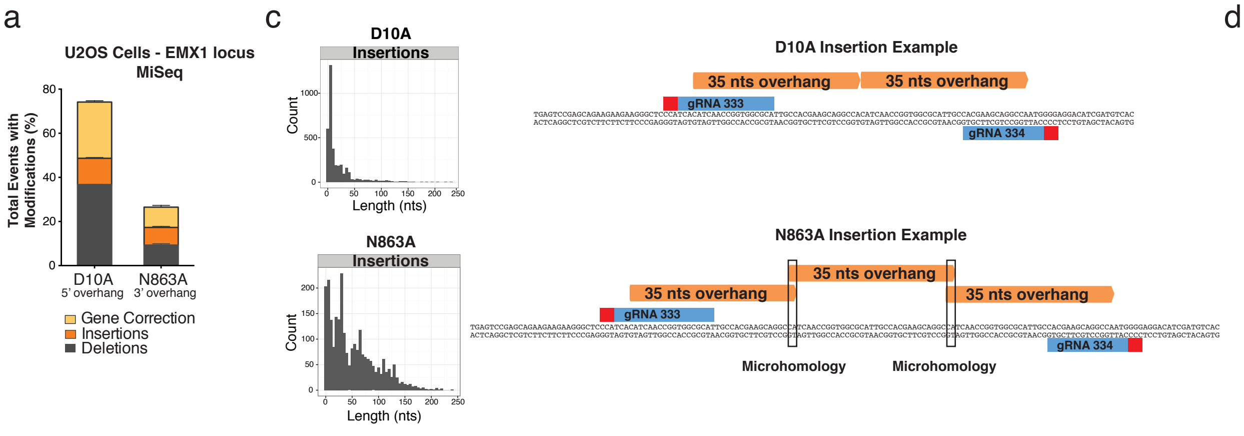

d

	D10A	N863A	p-value (D10A v. N863A)
# Insertions with overhang duplication (% of all insertions)	75 (20%)	6 (1%)	p<0.0001
# Insertions with micorhomology usage (% of all insertions)	7 (2%)	86 (13%)	p<0.0001
# Insertions analyzed	377	654	

p value: Fisher's exact test

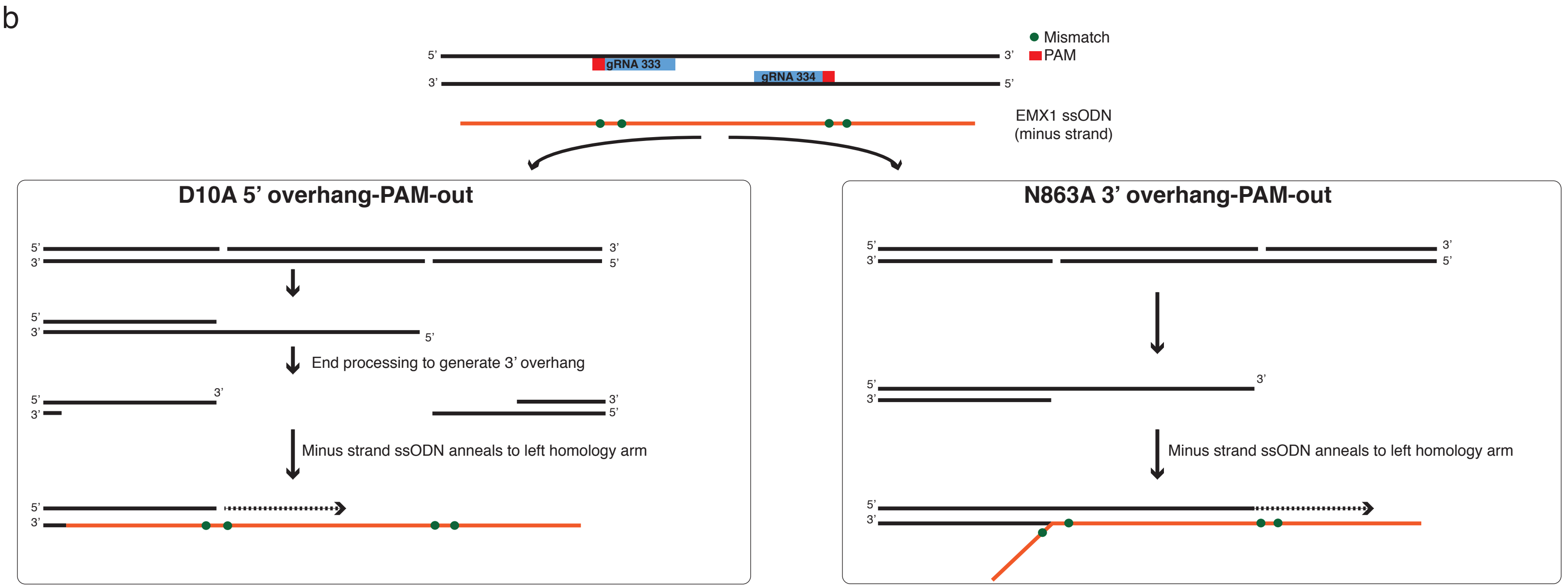

Supplementary Figure 6. Repair Distribution is Conserved at EMX1 Locus.

a. Bar graphs showing the overall modification frequency separated into deletions, insertions, and gene correction events for either D10A Cas9 or N863A Cas9 with two gRNAs targeting the EMX1 locus in U2OS cells. Data are represented as mean +/- SEM. Sequencing was performed using Illumina MiSeq.

b. Schematic of ssODN used for EMX1 dual nicking experiment. Mismatches with respect to EMX1 reference sequence are displayed as green dots.

c. Top: Histogram plots depicting insertion length after dual nicking at the EMX1 locus with D10A Cas9, along with an example of an insertion harboring an overhang duplication. Bottom: Histogram plots depicting insertion length after dual nicking at the EMX1 locus with N863A Cas9, along with an example of an insertion of overhang sequence that contains evidence of microhomology usage.

d. Table showing the percentages of insertions with overhang duplications or of insertions that display evidence of microhomology usage for either D10A or N863A Cas9 dual nicking at the EMX1 locus in U2OS cells. Fisher's exact test was performed to determine p-value. Sequencing was performed using Illumina MiSeq.

Supplementary Figure 7

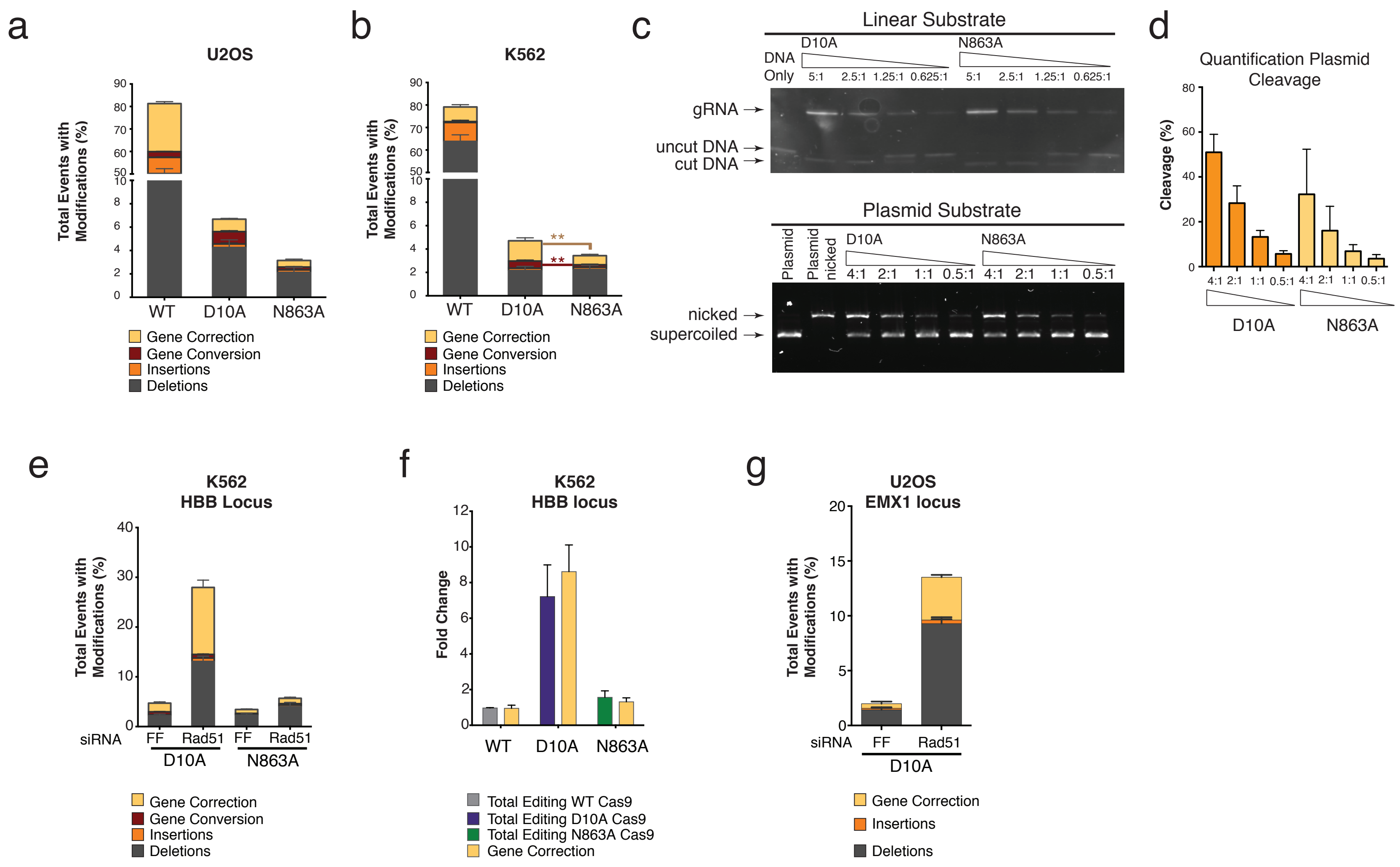

Supplementary Figure 7. Characterization of Repair Pathway Engagement of Cas9 Variant-Induced Single Nicks.

a. Frequency of deletions, insertions, gene conversion and gene correction of U2OS cells nucleofected with WT Cas9, D10A Cas9 and N863A Cas9 and gRNA 8. Sequencing was performed using an Illumina MiSeq. Three independent experiments. Data are represented as mean +/- SEM.
b. Frequency of deletions, insertions, gene conversion and gene correction of K562 cells nucleofected with WT Cas9, D10A Cas9 and N863A Cas9 and gRNA 8. Sequencing was performed using an Illumina MiSeq. Three independent experiments. Data are represented as mean +/- SEM. The p-values for the difference in gene correction ($p=0.0098$) and gene conversion frequency ($p=0.0056$) were calculated using the two-tailed Student's t-test and are indicated as * in the graph.
c. Top: Urea PAGE gel (15%) displaying cutting efficiency of decreasing amounts of D10A and gRNA8 or N863A and gRNA 8 complexes with linear double stranded DNA. Cleavage products are indicated. Bottom: Representative agarose gel of decreasing amounts of D10A and gRNA8 or N863A and gRNA 8 complexes with plasmid DNA.
d. Quantification of cleavage of three independent plasmid cleavage experiments. Bars are mean +/- SEM.
e. Frequency of deletions, insertions, gene conversion and gene correction observed after K562 cells were nucleofected with either D10A Cas9 or N863A Cas9 and gRNA 8 and siRNAs against firefly luciferase (FF) or RAD51. Sequencing was performed using MiSeq. Data are represented as mean +/- SEM.
f. Bar graph of fold change in the rates of total editing events and gene correction for the WT, D10A and N863A Cas9 mutants of cells treated with and siRNA against RAD51 relative to FF treated control. Three independent experiments. Data are represented as mean +/- SEM
g. Frequency of deletions, insertions and gene correction observed after U2OS cells were nucleofected with D10A Cas9 and gRNA 335 targeting the EMX1 locus and siRNAs against firefly luciferase (FF) or RAD51. Sequencing was performed using MiSeq. Data are represented as mean +/- SEM.

Supplementary Figure 8

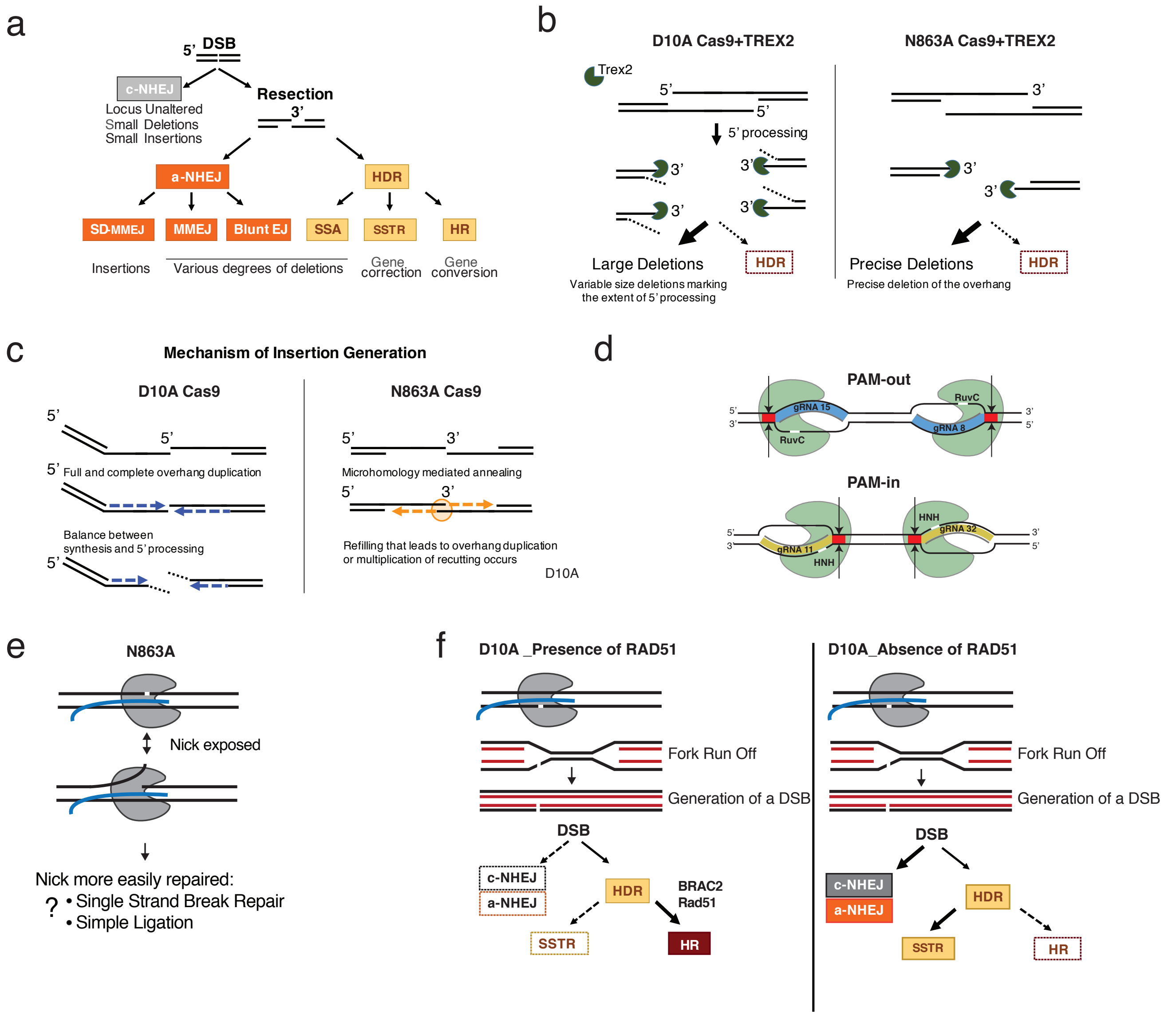

Supplementary Figure 8. Models

a. Model depicting the different possible repair outcomes after the formation of a DSB. C- NHEJ: Canonical Non-Homologous End Joining; A-NHEJ: Alternative-Non Homologous end joining, SD-MMEJ: Synthesis Dependent Microhomology Mediated End Joining, MMEJ: Microhomology Mediated End Joining; Blunt-EJ: Blunt End Joining; HDR: Homology Directed Repair; SSA: Single Strand Annealing; SSTR: Single Strand Template Repair, HR: Homologous Recombination.
b. Model depicting the transitions for D10A or N863A Cas9-induced paired nick lesions in the presence of TREX2.
c. Model depicting the generation of insertions for D10A or N863A-induced dual nick lesions.
d. Model depicting topological strains in PAM-in or PAM-out configurations. Arrows indicate the double-stranded DNA configuration of the PAM.
e. Model depicting the different repair possibilities of an exposed N863A Cas9-induced nicks at the non-target strand.
f. Model depicting the possible role of HR in processing of nicks that escaped single strand break repair in the presence and absence of RAD51.

Supplementary Information

Supplementary Table 1. List of Donor Sequences:

Donor Name	Donor Sequence
**High Mismatch Donor – ssODN minus strand**	TGCTTCTGACACAACTGTGTTCACTAGCAACCTCAAACAGACACCATG<br>GTGCATCTGACTCGTGTGGAGAAGTCGGCCGTTACTGCCCTGCAGG<br>GCAAGCTTAACGTGGATGAAGTTCGTGGTGAGGCCCTGGGCAGGTT<br>GGTATCAAGGTTACAAGACAGGTTTAAGGAGACCAATAG
**No mismatch Donor – ssODN minus strand**	AAAAGTCAGGGCAGAGCCATCTATTGCTTACATTTGCTTCTGACACAA<br>CTGTGTTCACTAGCAACCTCAAACAGACACCATGGTGCATCTGACTC<br>GTGTGGAGAAGTCTGCCGTTACTGCCCTGTGGGGCAAGGTGAACGT<br>GGATGAAGTTGGTGGTGAGGCCCTGGGCAGGTTGGTATCAAGGTTAC<br>AAGACAGGTTTA
**Sequence of Donor AHB_DonorPlasmid_3 (500 bp plasmid_no internal_mutations)**	TGAGCCTTCACCTTAGGGTTGCCCATAACAGCATCAGGAGTGGACAG<br>ATCCCCAAAGGACTCAAAGAACCTCTGGGTCCAAGGGTAGACCACCA<br>GCAGCCTAAGGGTGGGAAAATAGACCAATAGGCAGAGAGAGTCAGT<br>GCCTATCAGAAACCCAAGAGTCTTCTCTGTCTCCACATGCCCAGTTTC<br>TATTGGTCTCCTTAAACCTGTCTTGTAACCTTGATACCAACCTGCCCA<br>GGGCCTCACCATGAACTTCATCCACGTTCACCTTGCCCCACAGGGCA<br>GTAACGGCAGACTTCTCCTCACTAGTCAGATGCACCATGGTGTCTGT<br>TTGAGGTTGCTAGTGAACACAGTTGTGTCAGAAGCAAATGTAAGCAAT<br>AGATGGCTCTGCCCTGACTTTTATGCCCAGCCCTGGCTCCTGCCCTC<br>CCTGCTCCTGGGAGTAGATTGGCCAACCCTAGGGTGTGGCTCCACA<br>GGGTGAGGTCTAAGTGATGACAGCCGTACCTGTCCTTGGCTCTTCTG<br>GCACTGGCTTAGGAGTTGGACTTCAAACCCTC
**ssODN used for gRNA pair 333+334 at EMX1 locus (333+334 ssODN (minus))**	TGGCCAGCAGCAAGCAGCACTCTGCCCTCGTGGGTTTGTGGTTGCC<br>CACCCTAGTCATTGGAGGTGACATCGATGTACTCTCCATTGGCCTGC<br>TTCGTGGCAATGCGCCACCGGTTGAGGTGATGTGAGCCCTTCTTCTT<br>CTGCTCGGACTCAGGCCCTTCCTCCTCCAGCTTCTGCCGTTTGTACT<br>TTG
**ssODN used for gRNA 335 at EMX1 locus (335+336 ssODN (minus))**	GAGTCCAGCTTGGGCCCACGCAGGGGCCTGGCCAGCAGCAAGCAG<br>CACTCTGCCCTCGTGGGTTTGTGGTTGCGCACTCTAGTCATTGGAGG<br>TGACATCGATGTCCTCCGCATTGTCCTGCTTCGTGGCAATGCGCCAC<br>CGGTTGATGTGATGGGAGCCCTTCTTCTTCTGCTCGGACTCAGGCCC<br>TTCC

Supplementary Table 2. gRNA Sequences

Name	sequences	Length (bp)
S.pyogenes TRACR	GTTTTAGAGCTAGAAATAGCAAGTTAAAATAAGGCTAGTCCG TTATCAACTTGAAAAAGTGGCACCGAGTCGGTGCTTTTTTT	83
HBB- Guide RNA 8	GTAACGGCAGACTTCTCCTC	20
HBB- Guide RNA 15	AAGGTGAACGTGGATGAAGT	20
HBB- Guide RNA 19	CCTGTGGGGCAAGGTGAACG	20
HBB- Guide RNA 21	TGAAGTTGGTGGTGAGGCCC	20
HBB- Guide RNA 32	CATGGTGCATCTGACTCCTG	20
HBB- Guide RNA 11	CACGTTCACCTTGCCCCACA	20
EMX-Guide 333	TGCGCCACCGGTTGATGTGA	20
EMX-Guide 334	CACGAAGCAGGCCAATGGGG	20
EMX-Guide 335	GACATCGATGTCCTCCCCAT	20

Supplementary Table 3. Primer Sequences

**Name**	**Primer Sequence**
F1_HBB	5’-AGGCCATCACTAAAGGCACC-3’
R_HBB	5’-TAAGCCAGTGCCAGAAGAGC-3’
HBB_primer_1_3F	5’-GGAAAGAAAACATCAAGCGTCCCATA-3’
HBB_primer_1_3R	5’-GGCTGAGGGTTTGAAGTCCAAC-3’
HBD_primer_F	5’-CAGCCCAAGGGACAGAGAGT-3’
HBD_primer_R	5’-GGGCAGAGTCGACTGTTGC-3’
HBB_Miseq_1F	5’-CCATCTCATCCCTGCGTGTCTCCGACCACCAGCAGCCTAAGG-3’
HBB_Miseq_1R	5’-CCTCTCTATGGGCAGTCGGTGATGGCCATCTATTGCTTACATTTGCT-3’
AHB_MiSeq_340	5’-CCATCTCATCCCTGCGTGTCTCCAGGTGAAGGTGTGGTTCCAG-3’
AHB_MiSeq_341	5’-CCTCTCTATGGGCAGTCGGTGATGCAGGGAGTGGCCAGAGTC-3’

Supplementary Note 1.

The apparent overall modification frequency for the N863A-induced nicks is lower than in previous examples (**Fig. 1a and Fig. 5a**). While all previous experiments were performed using Sanger sequencing as a readout, this experiment was performed using high throughput sequencing of the *HBB* amplicon with an Illumina MiSeq. To see whether the sequencing methodology and subsequent analysis would affect the repair distribution, we subjected samples that we previously analyzed with Sanger sequencing to MiSeq sequencing (**Supplementary Fig. 5d**). We noticed that particularly insertions in the N863A dual nick condition but not the D10A dual nick condition are underrepresented by MiSeq analysis compared to Sanger sequencing, which we have previously validated to be a representative way of measuring repair frequencies using single cell clone analysis (**Supplementary Fig. 1d**). There are several differences between these methodologies that could contribute to the discrepancy in overall modification frequency that we observe for the N863A dual nickases: (1) Two rounds of PCR were performed for library preparation (see Methods), which can result in increased chances for PCR bias towards small products, underrepresenting larger products (repair events with insertions), (2) Size selection when preparing the library for sequencing would disfavor products with long insertions, (3) Amplification of the DNA on the slides of the MiSeq that allows the generation of sequence will bias against larger insertions, and (4) Shorter Miseq reads do not allow sequencing through the entire insertion. All these differences would result in the underestimation of long insertions (**Supplementary Fig. 5d**), which would have a higher impact on the N863A dual nick condition, as there we observe the occurrence of significantly longer insertions than in the D10A dual nick condition While we underestimate the overall frequency of the N863A-induced insertions, the distributions of the insertion lengths are similar for both sequencing methods (**Supplementary Fig 5e**). Moreover, we noticed that GC was underrepresented in both D10A and N863A conditions, which is likely due to the asymmetrical distribution of GC events (30% of GC events are beyond the boundaries of the nicks), resulting in an underestimation of events when using detection methods that rely on smaller amplicons, that would not prime in a region in which GC conversion tracts are present.

Supplementary Methods

Single Cell Cloning

250K U2OS cells per condition were nucleofected using Lonza 4D Nucleofector (AAF-1002B 4D-Nucleofector™ Core unit, AAF-1002X 4D-Nucleofector™ X unit). The cells were plated in 6 well dishes. For each of the conditions, 0.3 cells/well were plated in 3X 96 well plates. After 3 weeks, the 96 well plates were scored by eye via microscope for single colony formations. More than 80 clones were picked for each condition and analyzed by TOPO cloning the PCR products of the amplified locus (as described above). The competent cells were platted on LB agar containing ampicillin plates (Molecular Devices # NC0183882) and 6 colonies were requested to be picked for each subclone.

Western Blotting

The cells were lysed using 1X RIPA (RIPA-500 Boston BioProducts 5X BP-115-5X) with 1X phosphatase, 1X protease inhibitor (Roche 04-906-837-001 and 05-892-7911-001), 1 mM MgCl and 100 U/mL benzonase (EMD Millipore #71205). The lysates were incubated on ice for 30 minutes and were then centrifuged at 15000g for 10 minutes at 4°C. The protein quantification was done by BCA protein assay (ThermoFisher Scientific #23225). All samples were boiled using LDS sample buffer and DTT (Life Technologies Ref NP0007 and Ref NP0009) for 10 minutes at 70°C. For BRCA2 samples, 30 µg of total protein was run on 3-8% Tris-Acetate gels (Life Technologies EA0375BOX) for 1 hour at 150V. For the Rad51 samples, 15 µg total protein was run on a 4-12% Bis-tris gel (Life Technologies NP0322BOX) for 75 minutes at 150V. All gels were transferred with 1X transfer buffer (Boston Bioproducts BP-190) with 10% Methanol for 1 hour at 100V at 4°C. All gels were transferred onto 0.2 µM nitrocellulose membrane. For BRCA2 blots, 5% milk dissolved in PBS was used for blocking, primary and secondary incubations. For the Rad51 blots, 5% fish gelatin (Sigma-Aldrich Gelatin G7041-500G) dissolved in 1X PBS-T was used for blocking and 2% fish gelatin was used for primary and secondary antibody incubations. The blots were all blocked for one hour at room temperature. Primary antibody was incubated over night at 4°C with the following dilutions: 1:1000 Calbiochem BRCA2 OP95 and 1:250 Santa Cruz Rad51 (H-92) sc-8349, 1:250 Trex2 Abnova PAB16682, 1:2000 Cas9 (Diagenode). The following was used

to probe for loading controls: 1:5000 Cell Signaling Beta-actin 3700S and 1:3000 Sigma V9131-.2ML Monoclonal Anti-vinculin antibody produced in mouse. 1:20000 GE Amersham ECL Anti-mouse IgG, Horseradish Peroxidase linked whole antibody (from sheep) 1mL NA931V and 1:20000 Jackson ImmunoResearch Goat Anti-Rabbit IgG (H+L) 111-035-003 was used for secondary antibody incubation which was done for 1 hour at room temperature. 1X PBS-T was used for the 10 minute washes 3X before and after secondary antibody incubation. BRCA2 blots were developed using FEMTO substrate (Thermo Scientific SuperSignal West FEMTO Prod #34095); the blots were incubated for 5 minutes at room temperature. Rad51, actin and vinculin blots were developed using ECL substrate (Perkin Elmer Kit NEL105001EA); the blots were incubated for 1 minute at room temperature. Blots were imaged using Bio-Rad ChemiDoc System.

In-vitro **Cutting Assay**

10uL reactions in 50mM Hepes pH7.5, 150mM NaCl, 2mM $MgCl_2$ containing 50nM of dsDNA oligo of 45bp length with varying amounts of RNP:DNA ratio as follows 5:1, 2.5:1, 1.25:1, 0.625:1 were incubated at 37°C for 15 minutes. Similarly, 10µL reactions in 50mM Hepes pH7.5, 150mM NaCl, 2mM $MgCl_2$ containing 20nM of gel-purified supercoiled plasmid DNA with varying amounts of RNP:DNA molar ratio (4:1, 2:1, 1:1, 0.5:1) were incubated at 37°C for 20 minutes. The reactions were quenched by addition of 1uL 0.5M EDTA, 1uL 40ug/ul Proteinase K and incubated at 50°C for 15 minutes. For the linear dsDNA cutting assay, 3µL of sample was removed and mixed with 3uL of TBE Urea sample buffer (Bio-Rad #1610768) and heated at 80°C for 10 minutes. All material was loaded onto a 15% Mini-PROTEAN TBE-Urea Gel (Bio-rad #4566055) and run at 200V for 45 minutes. The gel was incubated with SYBR Safe DNA Gel Stain (ThermoFisher #S33102) for imaging of the nucleic acid bands with a Bio-Rad Chemi-Doc station. For the plasmid cleavage assay 8uL of each sample mixed with DNA loading buffer was run on a 0.7% agarose gel containing SYBR Safe DNA Gel Stain (ThermoFisher #S33102) at 150V for 1 hour. Subsequent imaging for nucleic acid bands was performed on a Bio-Rad Chemi-Doc station. Quantification of cutting efficiency was performed using ImageJ.
